# Temporal Dynamics of Abundance and Composition of Nitrogen-Fixing Communities across Agricultural Soils

**DOI:** 10.1371/journal.pone.0074500

**Published:** 2013-09-13

**Authors:** Michele C. Pereira e Silva, Brigitte Schloter-Hai, Michael Schloter, Jan Dirk van Elsas, Joana Falcão Salles

**Affiliations:** 1 Department of Microbial Ecology, University of Groningen, Groningen, The Netherlands; 2 Research Unit for Environmental Genomics, Helmhotz Zentrum München, Neuherberg, Germany; Dowling College, United States of America

## Abstract

**Background:**

Despite the fact that the fixation of nitrogen is one of the most significant nutrient processes in the terrestrial ecosystem, a thorough study of the spatial and temporal patterns in the abundance and distribution of N-fixing communities has been missing so far.

**Methodology/Principal Findings:**

In order to understand the dynamics of diazotrophic communities and their resilience to external changes, we quantified the abundance and characterized the bacterial community structures based on the *nif*H gene, using real-time PCR, PCR-DGGE and 454-pyrosequencing, across four representative Dutch soils during one growing season. In general, higher *nifH* gene copy numbers were observed in soils with higher pH than in those with lower pH, but lower numbers were related to increased nitrate and ammonium levels. Results from *nif*H gene pyrosequencing confirmed the observed PCR-DGGE patterns, which indicated that the N fixers are highly dynamic across time, shifting around 60%. Forward selection on CCA analysis identified N availability as the main driver of these variations, as well as of the evenness of the communities, leading to very unequal communities. Moreover, deep sequencing of the *nifH* gene revealed that sandy soils (B and D) had the lowest percentage of shared OTUs across time, compared with clayey soils (G and K), indicating the presence of a community under constant change. Cosmopolitan *nif*H species (present throughout the season) were affiliated with *Bradyrhizobium* , *Azospirillum* and *Methylocistis*, whereas other species increased their abundances progressively over time, when appropriate conditions were met, as was notably the case for *Paenibacilus and Burkholderia.*

**Conclusions:**

Our study provides the first in-depth pyrosequencing analysis of the N-fixing community at both spatial and temporal scales, providing insights into the cosmopolitan and specific portions of the nitrogen fixing bacterial communities in soil.

## Introduction

The nitrogen cycle is one of the most significant nutrient cycles in the terrestrial ecosystem. Among its processes, biological nitrogen fixation (BNF), the reduction of atmospheric N_2_ gas to biologically available ammonium, is a key one, as it replenishes the pool of reduced nitrogen that is lost to the atmosphere via anaerobic ammonium oxidation and denitrification [1]. BNF is widely distributed across bacterial and archaeal taxa [2], being performed by a highly diverse group of microorganisms. These diazotrophic organisms harbor the *nif*H gene, which encodes part of the nitrogenase enzyme. This gene is considered to be a suitable marker for studying the diversity and composition of N fixers without the need of microbial cultivation [3,4]. In traditional agricultural fields, nitrogen input may rely to some extent on biological N fixation, which occurs primarily (but not exclusively) by diazotrophic bacteria in symbiosis with legumes [5]. It has been suggested that symbiotic N-fixers can fix up to 150 kg N/ha/year in some ecosystems [6]. By contrast, estimates of the contribution of free-living nitrogen fixation are much lower (0 to 60 kg N/ha/year) [7]. However, these can also be significant and even critical under certain conditions, depending on the environment [8-10].

It is known that the local environment in which soil organisms coexist often changes with time [11], which influences how they structure themselves and perform their functions. Furthermore, global change is expected to severely affect N turnover in soils [12], impacting processes of the nitrogen cycle to an unknown extent. In this sense, several classes of nitrogen-fixing bacteria might be affected, as these are known to be sensitive to perturbation [13,14]. In general, fluctuations in local conditions affect the soil microbial community structure and composition [15,16] at different temporal and spatial scales. For instance, it has been found that the temporal variation in nitrogen fixation rates within ecosystems may vary up to 50% across seasons [17-19]. These temporal variations are likely driven by fluctuations in precipitation [20] and temperature [21]. Agricultural practices, such as fertilization and ploughing, also play major roles as determinants of bacterial community structure in soil [22-24]. In this context, several environmental factors have been suggested to influence N fixation in soils, including soil moisture, oxygen, pH, carbon quantity and quality, nitrogen availability [3], soil texture and aggregate size [25] and clay content [8]. Changes in nutrient availability also alter the rates of nitrogen fixation through changes in community composition [3,26,27]. For instance, the expression of *nif*H gene was found to be down-regulated under low P conditions [28]. Moreover, Lindsay et al. [27] observed a negative correlation between *nif*H gene abundance and high N concentrations in a managed ecosystem.

Understanding temporal and spatial patterns in the abundance and distribution of communities has been a fundamental goal of ecology. In order to understand the functioning of microbial communities and their resilience to external changes, a key issue is to assess the community composition, quantify individual microbial population sizes and study fluctuations thereof [29,30]. The inclusion of the so-called rare micro biosphere in ecological analyses is challenging in the face of the technical noise in pyrosequencing data [31,32], and such information has been so far missing for nitrogen fixing communities. Therefore, the aim of this study was to provide a thorough description of the natural variation of diazotrophic organisms in a wide range of soils. To this end, we characterized the spatio-temporal variability of *nif*H-containing microorganisms in agricultural soils, as measured by changes in PCR-DGGE fingerprints, real-time PCR and *nif*H-gene pyrosequencing, and related this variability to environmental conditions. These analyses were performed for four different soils at three times over one growing season, i.e. April, June and October 2010. Considering the sensitivity of diazotrophic organisms to N fertilization, we hypothesized that their abundance and diversity would be lower at the beginning of the season, when fertilizers are applied, compared to October. Moreover, due to the strong effect of soil type on microbial communities [4,33] we hypothesized that these differences will be more relevant in clayey than in sandy soils.

## Materials and Methods

### Sampling sites

Four soils (B, D, G and K) from different sites in the Netherlands were sampled three times in 2010, April (after seedling), June (before flowering) and October (senescence stage). The fields are used for potato cropping and are under agricultural rotation regime with non-leguminous crops. Information on land use, fertilization scheme and location is available (Table 1). The soils were chosen to represent two different soil types (clay: G and K vs. sand; B and D) and chemical properties (Table 1 and Table S1). Bulk soil samples (4 replicates per soil; 0.5kg per replicate) were collected in plastic bags and thoroughly homogenized before further processing in the lab. A 100-g subsample was used for measuring ammonia oxidizing enzyme activity, next to molecular and soil chemical properties.

**Table 1:** Sampling locations, environmental and biological data.

Sampling Location	Buinen (B)	Droevendaal (D)	Kollummerwaard (K)	Grebbedijk (G)
Soil type	Sandy loam	Sandy loam	Clayey	Clayey
Land use	Agricultural	Agricultural	Agricultural	Agricultural
Sampling coordinates	52°55’386"N 006°49’217"S	51°59’551"N 005º39’608"S	53°19’507"N 006°16’351"S	51°57’349"N 005º38’086"S
Fertilization scheme/date	KAS (212Kg N/ ha) April and June	NPK (200Kg N/ha) March	KAS (150Kg N/ha) March and April	KAS (156Kg N/ha) March and April
pH	4.40±0.02	4.93±0.02	7.40±0.05	7.20±0.02
N-NO_3_^-^ (mg/kg)	47.51±0.37	60.53±1.66	24.60±1.72	29.93±1.15
N-NH_4_^+^ (mg/kg)	9.23±0.74	14.18±1.41	8.40±0.75	15.13±2.35
OM (%)	3.96±0.72	2.91±0.24	4.24±0.14	5.47±0.22
Water content (%)	11.20±0.58	11.63±1.62	19.30±1.40	19.60±1.86
*nif*H-gene abund (x 10^5^ gdw^-1^).	0.72±0.28	1.15±0.43	17.0±8.13	3.10±0.94
Bacterial 16S rRNA gene abund (x 10^10^ gdw^-1^).	1.74±0.21	1.68±0.13	1.98±0.15	1.98±0.13

Values of environmental and biological data are average of each soil across the three sampling times (mean ± sd), KAS = calcium, ammonium, nitrate; NPK = nitrate, phosphorus, calcium

### Soil chemical analysis

The soil pH was measured after shaking a soil/water (1:2, w:v) suspension for 30 min (Hanna Instruments BV, The Netherlands). Gravimetric soil moisture contents were determined by comparison of fresh and dried (105°C; 24h) weight of samples. Organic matter (OM) content was calculated as the difference between the initial and final sample weights of dried soil measured after 4 hours at 550°C. Nitrate (N-NO_3_^-^) and ammonium (N-NH_4_^+^) were determined in CaCl_2_ extracts by a colorimetric method using the commercial kits Nanocolor Nitrat50 (detection limit, 0.3 mg N kg^-1^ dry weight, Macherey-Nagel, Germany) and Ammonium3 (detection limit, 0.04 mg N kg^-1^ dry weight; Macherey-Nagel, Germany) according to Töwe et al. [34].

### DNA extraction

DNA was extracted from 0.5 g soil using the FastDNA SPIN Kit for Soil (MP Biomedicals) according to manufacturer’s protocol. Extracted DNA was then precipitated and concentrated with cold ethanol to remove impurities. DNA concentrations were determined using the Quant-iT™ PicoGreen® dsDNA Reagent (Invitrogen, Germany) according to the manufacturer’s protocol using a spectrofluorimeter (Spectramax Gemini, Germany). The quality of extracted DNA was estimated by running on agarose gel based on the degree of DNA shearing (average molecular size) as well as the amounts of coextracted compounds.

### PCR-DGGE analysis

PCR reactions for DGGE analysis were performed targeting bacterial 16S rRNA gene as well as the *nif*H gene, as a proxy of nitrogen-fixing community. Bacterial 16S rRNA gene was PCR amplified using the forward primer F968 [35] with a GC-clamp attached to 5’ and the universal primer R1401.1b [36]. PCR of the *nif*H gene was performed using a nested PCR according to Diallo et al. [37]. PCR reactions and cycling conditions are described in Table S2. DGGE profiles were generated with the Ingeny Phor-U system (Ingeny International, The Netherlands). The PCR products (300 ng/lane) were loaded onto 6% (w/v) polyacrylamide gels, with a denaturant gradient of 45-65% for total bacterial community and 40-65% for nitrogen-fixing community (100% denaturant corresponded to 7 M urea and 40% (v/v) deionized formamide). Electrophoresis was performed at a constant voltage of 100 V for 16 h at 60°C. The gels were stained for 60 min in 0,5x TAE buffer with SYBR Gold (final concentration 0,5 µg/liter; Invitrogen, The Netherlands). Images of the gels were obtained with Imagemaster VDS (Amersham Biosciences, United Kingdom).

### Quantitative PCR

The gene abundances in the soil samples were quantified using quantitative PCR (qPCR). For the *nif*H gene, the primers FPGH19 [38] and PolR [39] were used according to Pereira e Silva et al. [4]. To be able to compare the fluctuations of the diazotrophic community, we also quantified the bacterial 16S rRNA gene, using specific primers for V5-V6 region of bacteria 16SFP/ 16SRP [40]. Cycling programs and primer sequences are found in Table S2. Standard curves were generated from serial dilutions of plasmid containing cloned partial *nif*H gene from *Bradyrhizobium* *liaoginense* , and partial bacterial 16S rRNA gene from *Burkholderia* *terrae* BS001, from 10^6^ to 10^2^ gene copy numbers/µl. Absolute quantification was carried out twice from each of the four soil replicates on the ABI Prism 7300 Cycler (Applied Biosystems, Germany). The specificity of the amplification products was confirmed by melting-curve analysis, and the expected sizes of the amplified fragments were checked in 1.5% agarose gel stained with ethidium bromide. Possible inhibitory effect of co-extracted humid compounds was checked by spiking standard concentrations with serial dilutions of soil samples. No severe inhibition was observed at the working dilutions. The qPCR efficiency (E) was calculated according to the equation E = 10^[-1/slope]^ [41] and was 1.94 (bacterial 16S rRNA gene) and 1.92 (*nif*H gene). The R^2^ of all these standards was higher than 0.98.

### Deep-sequencing and bioinformatics analysis of nifH gene

The diversity of nitrogen-fixing bacteria in the four agricultural soils was investigated through barcoded pyrosequencing. The total community DNA was amplified with the *nifH* gene-specific primers PolF/PolR [39] and RoeschF/ RoeschR [42] in a nested approach using the FastStart High Fidelity PCR system and PCR Nucleotide Mix (Roche Diagnostics GmbH, Germany). PCR conditions and primer sequences are presented in Tables S3 and S4. Triplicate PCR amplifications were performed on each soil DNA template and pooled. Primer dimers were removed by electrophoresis of PCR products on agarose gel, excision, and purification using Qiaquick PCR purification Kit (Qiagen). For pyrosequencing, adapters and sample-specific tags were added to the samples using custom primers in an additional PCR amplification of 20 cycles using the same PCR conditions. Amplicons were further purified with Agencourt AMpure Beads XP (Beckman Coulter, Germany) and pooled in an equimolar ratio as specified by Roche. Sequencing from 5’ (forward) and 3’ (reverse) ends of amplicons was performed. Emulsion PCR, emulsion breaking of DNA-enriched beads, and sequencing runs of the amplicon pools were performed on a second-generation pyrosequencer (454 GS FLX Titanium; Roche) using titanium reagents and titanium procedures as recommended by the manufacturer.

Quality filtering of the pyrosequencing reads was performed using the automatic amplicon pipeline of the GS Run Processor (Roche) to remove failed and low quality reads from raw data. Amplicon libraries of the nitrogenase gene (*nif*H) were explored using the FunGene Pipeline of RDP server (http://fungene.cme.msu/edu/FunGenePipeline) using the default settings. Primer sequences were trimmed and reads of low quality and shorter than 320 bp were removed. Filtered nucleotide sequences were translated into amino acid and clipped at 108 bp. All subsequent analyses were done on amino acid sequences. By targeting a protein-encoding gene, frame-shift errors caused by insertions or deletions of bases, can be identified [43]. Sequences were then visually inspected and sequences containing in-frame stop codon(s) were removed. The amino acid sequences were aligned in MUSCLE 3.8 [44].

Operational taxonomic units (OTUs) were then classified and rarefaction curves were constructed with DOTUR [45] using a 90% amino acid sequence similarity cutoff level [46,47]. Richness estimates and diversity indices were calculated for the total number of sequences as well as for subsets normalized to the same number of sequence depth, which were performed using the Perl script daisychopper.pl (available at http://www.genomics.ceh.ac.uk/ GeneSwytch/Tools.html). Phylogenetic analysis was performed for clustered sequences at 90% similarity using CD-HIT [48], and were so called here *abundant* (*core*) OTUs. *Rare* (satellite) species, in contrast to the abundant (core) ones, are defined here as the OTUs (clustered at 90% similarity cutoff) that were represented by less than 10 sequences in the whole dataset. The *nif*H representative sequences were blasted against a non-redundant protein sequence database using BLASTp, and used to build neighbor-joining trees in MEGA5 [49]. Normalized weighted UniFrac significance [50] was calculated and PCoA analysis was done to evaluate differences between the *nif*H-gene communities based on the phylogenetic trees obtained in MEGA5.

### Data analysis

Computer-assisted analysis of DGGE profiles was performed using the GelCompar software (Applied Maths, Belgium), to follow the structural changes on these communities over time. Similarity matrices were constructed based on Pearson’s correlation coefficient, and cluster analyses were done by unweighted pair group method with average linkages (UPGMA). The differences in soil chemical parameters and *nif*H gene abundances in the different soils over time were estimated with independent t-Tests. To test the influence of soil physicochemical parameters (environmental factors) on the community data, forward selection was used via canonical correspondence analysis (CCA) or redundant correspondence analysis (RDA), depending on the gradient length observed on DCA, using Canoco (version 4.0 for Windows, PRI, Wageningen, The Netherlands). In cases where gradient length was longer than 4.0, a unimodal method (CCA) was used. Otherwise, with gradients shorter than 4.0, RDA was chosen. For this, all parameters except pH were log_10_-transformed, and the community matrices (obtained from DGGE bands as well as from pyrosequencing) were square-root transformed. Furthermore, to test how species richness, evenness, gene abundance and the relative abundance of OTUs varied in relation to environmental variables, parametric Pearson correlation coefficients were calculated in SPSS v20.0 (SPSS Inc., USA). To visualize changes in most abundant and rare genera across samples, we constructed heatmaps and dendrograms with clustering based on Ward’s minimum variance and utilizing Euclidian distance calculation with scaling. The dendrograms are not phylogenetic but clustered based on the relative abundance among samples. Thus, more similar samples are more closely related. These analyses were done in the R package pheatmap (http://cran.r-project.org/web/packages/pheatmap/index.html).

### Data Availability

Sequences from this study were submitted to MG-RAST Metagenomic Analysis Server under the ID numbers 4524846.3, 4524847.3, 4524848.3, 4524849.3, 4524850.3, 4524851.3, 4524852.3, 4524853.3, 4524854.3, 4525982.3, 4525983.3 and 4525984.3.

## Results

### Seasonal variations of soil chemical properties

Soil pH, nitrate, ammonium and organic matter levels were determined in triplicate across all soil samples (Table 1). Individual values for each soil at each sampling time can be found in Table S1.

pH and OM levels were significantly higher (P < 0.05) in the clayey soils K and G than in the sandy ones B and D. In contrast, the levels of nitrate were higher in the sandy soils (B and D, 47.17 ± 0.37 and 60.53 ±1.66, respectively) than in the clayey ones (K and G, 29.93 ± 1.15 and 24.60 ± 1.72, respectively). The levels of ammonium also varied over the whole period: relatively low values were observed for all soils in October (on average 5.47 mg/kg ± 0.66) and higher levels in April and June (on average, 14.63 mg/kg ± 2.66 and 14.01 mg/kg ± 0.61, respectively).

### Size of total bacterial and N-fixing community over time as related to soil variables

**Figure 1:**
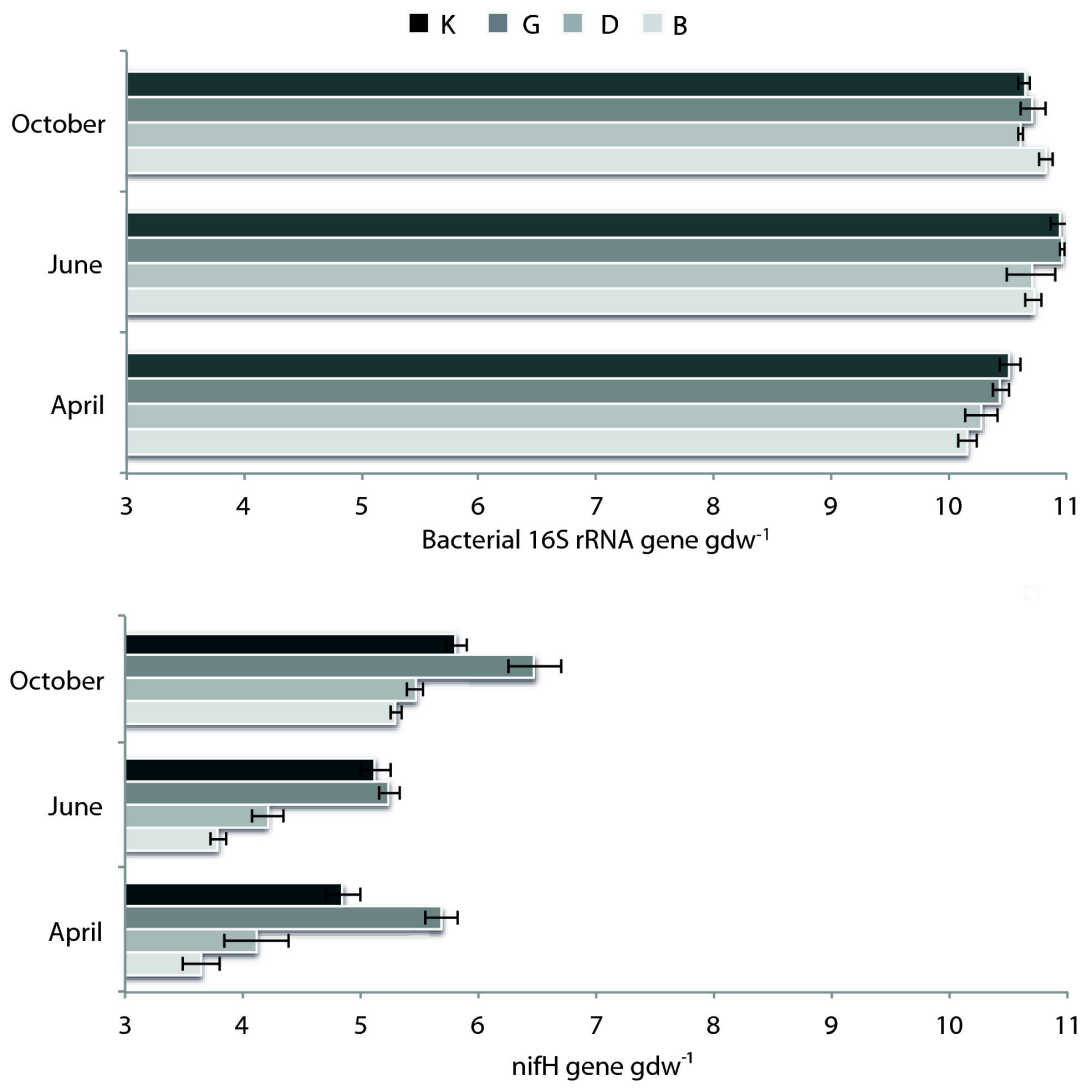
Changes in abundance of bacterial 16S rRNA and *nif*H genes. Soils B, D, K and G were collected in April, June and October of 2010. The copy numbers per gram of dry soil was estimated by real-time PCR. Soils: B, Buinen; D, Droevendaal; K, Kollumerwaard and G, Grebbedijk.

We quantified the abundance of the bacterial 16S rRNA gene to be able to draw conclusions regarding the fluctuations of the diazotrophic population. Total bacterial abundance showed small fluctuations from April to June but were quite stable from June to October, with copy numbers varying from 1.34 x 10^10^ gdw^-1^ in April to 1.36 x 10^11^gdw^-1^ in June (Figure 1). Moreover, no significant difference in bacterial abundance between sandy and clayey soils was observed. Regarding the N-fixing community, the overall (all soils) *nif*H gene copy numbers fluctuated from 5.30 x 10^3^ to 4.34 x 10^6^ gdw^-1^ over the growing season, being significantly higher (P< 0.05) in October (1.38 x 10^6^ gene copy numbers gdw^-1^) compared to April (1.69 x 10^5^ gene copy numbers gdw^-1^) and June (9.13 x 10^4^ gene copy numbers gdw^-1^) (Figure 1). Analysis per soil revealed that the clayey soils K and G had significantly higher *nif*H gene abundance at all sampling times (1.0 x 10^6^ gene copy numbers gdw^-1^ on average) compared to the sandy soils B and D (9.33 x 10^4^ gene copy numbers gdw^-1^ on average). Pearson’s product-moment correlations were calculated to test the influence of soil variables on bacterial 16S rRNA and *nif*H gene abundance. None of the soil parameters measured were able to explain the small variations in total bacterial abundance. However, nitrate (r = -0.543, P≤0.0001), ammonium (r = -0.565, P≤0.0001) and pH (0.645, P≤0.0001) were identified as the main drivers of variation in *nif*H gene abundance.

### Structure of the nifH gene carrying communities in relation to soil variables

**Figure 2:**
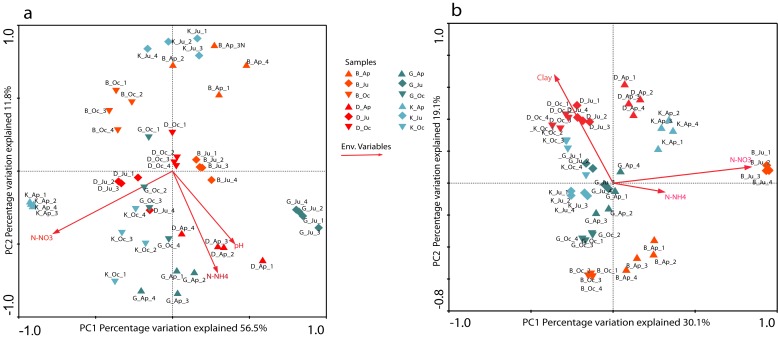
Changes in the structure of total bacterial and N-fixing communities from DGGE fingerprints across different soils and sampling times. Changes in the structure of bacterial (a) and N-fixing (b) communities and the influence of environmental parameters, as revealed by CCA. Only statistically significant environmental variables are shown. The number in each axis shows the percentage of total variation explained. The length of the corresponding arrows indicated the relative importance of the geochemical factor in explaining the variation in microbial profiles. Soil samples were analyzed in four replicates at each sampling time. B, Buinen; D, Droevendaal; K, Kollumerwaard and G, Grebbedijk; Ap, April; Ju, June; Oc, October.

In order to characterize the dynamics of the N-fixing communities through time, we performed PCR-DGGE analyses based on the bacterial 16S rRNA and *nif*H genes. Concerning the analysis of the resulting bacterial profiles, we observed similar number of bands in all soils, (approximately 41±1, 46±1, 44±1 and 41±1 for soil B, D, G and K respectively), and tended to decrease from April to June in all soils, increasing again from June to October (Figure S1). Forward selection on canonical correspondence analysis (CCA) (Figure 2A) revealed nitrate (10.3%, P = 0.032), ammonium (2.1%, P = 0.021) and pH (1.9%, P = 0.021) as main determinants of the structural changes in the bacterial community. DGGE analysis based on the *nif*H gene revealed similar results. Moreover, the number of bands tended to decrease from April to October for the sandy soils, but remained constant in the clay soils. Such number of bands were low and varied to a limited extent over time, with averages of 15±2, 12±1, 11±2 and 11±1 for soils B, D, K and G, respectively (Figure S1). Analysis of the Shannon diversity index values for the whole dataset (data not shown) indicated that the date of sampling had no significant effect on *nif*H gene diversity, except for soil G, which showed a significantly lower Shannon index in June as compared to April and October. Overall, the *nif*H gene-based profiles clustered into five groups at 38% similarity (Figure S2). The clustering reflected an increasing effect of sampling time on the N-fixing community structure. Forward selection on canonical correspondence analysis (CCA) revealed nitrate (28.6%, P = 0.04), clay content (24.4%, P = 0.048) and ammonium (16.1%, P = 0.003) as the main factors affecting this clustering (Figure 2B).

### Pyrosequencing-based assessment of nifH gene diversity

To further analyze the changes in the composition of the communities of N-fixing microorganisms in the four agricultural soils, the *nif*H gene was sequenced using pyrosequencing. To assess the effect of any technical biases, the forward and reverse reads were analyzed separately. In total, 91,015 reads were obtained. The total read numbers per sample are described in Table S5. These (transformed into amino acid sequences, average length of 108 amino acids) ranged from 1,921 to 5,892, corresponding to 40-120 OTUs (defined at a 10% sequence difference). The forward reads of the *nif*H amplicons yielded slightly more OTUs than did the reverse ones; however, the number of sequences was lower, indicating that the resolving power of the forward *nif*H reads was higher for diversity analysis than that of the reverse reads. In addition, the results obtained from the reverse reads showed similar trends to those obtained from forward ones. Therefore, we only present the results obtained from the forward reads.

**Figure 3:**
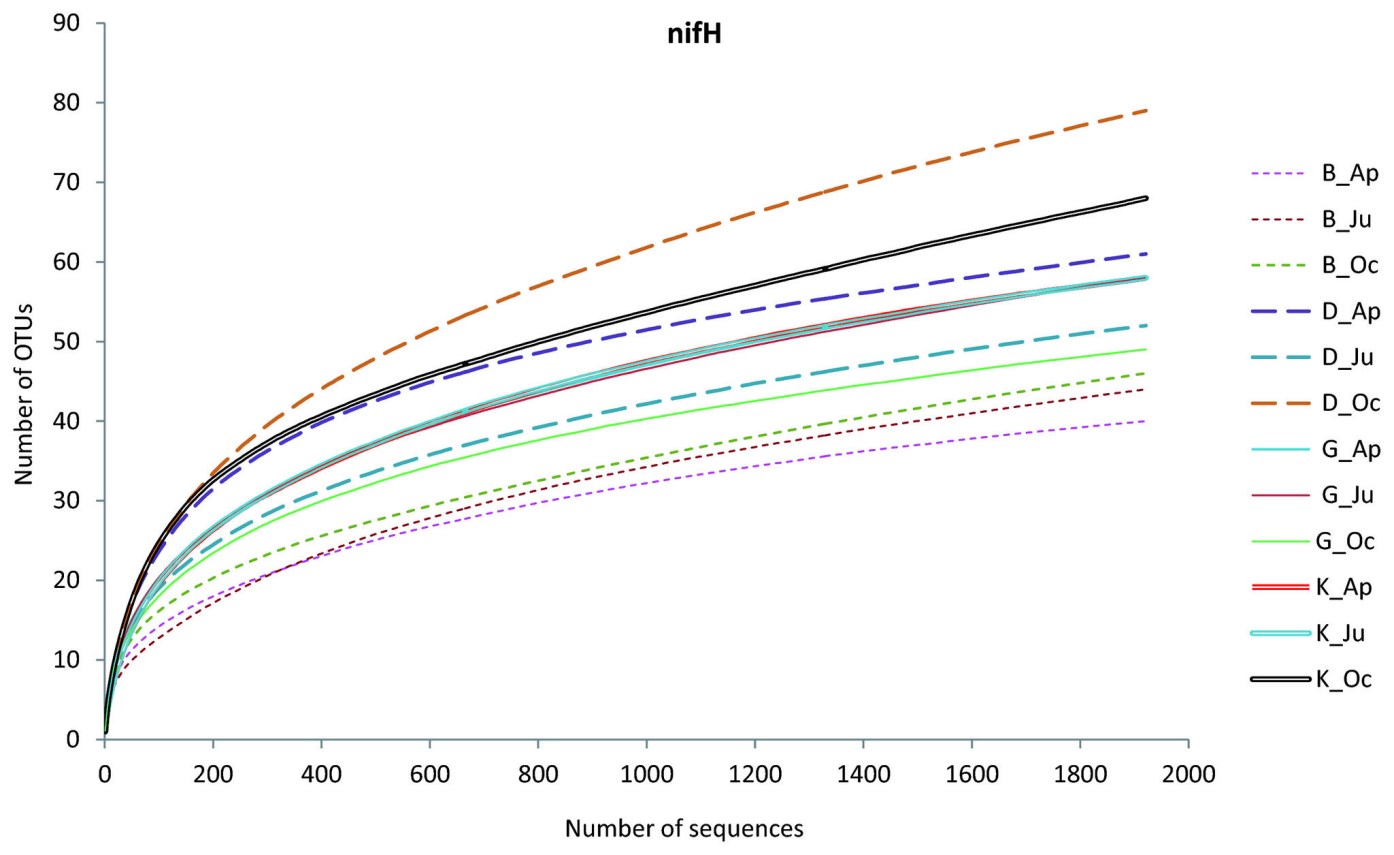
Rarefaction analysis of the diversities of *nif*H gene. Analyses of four soils across three sampling times after resampling of the sequences to the same depth (1921 sequences). The OTUs were classified at 90% similarity cutoff based on amino acid sequences. B, Buinen; D, Droevendaal; K, Kollumerwaard and G, Grebbedijk; Ap, April; Ju, June; Oc, October.

**Figure 4:**
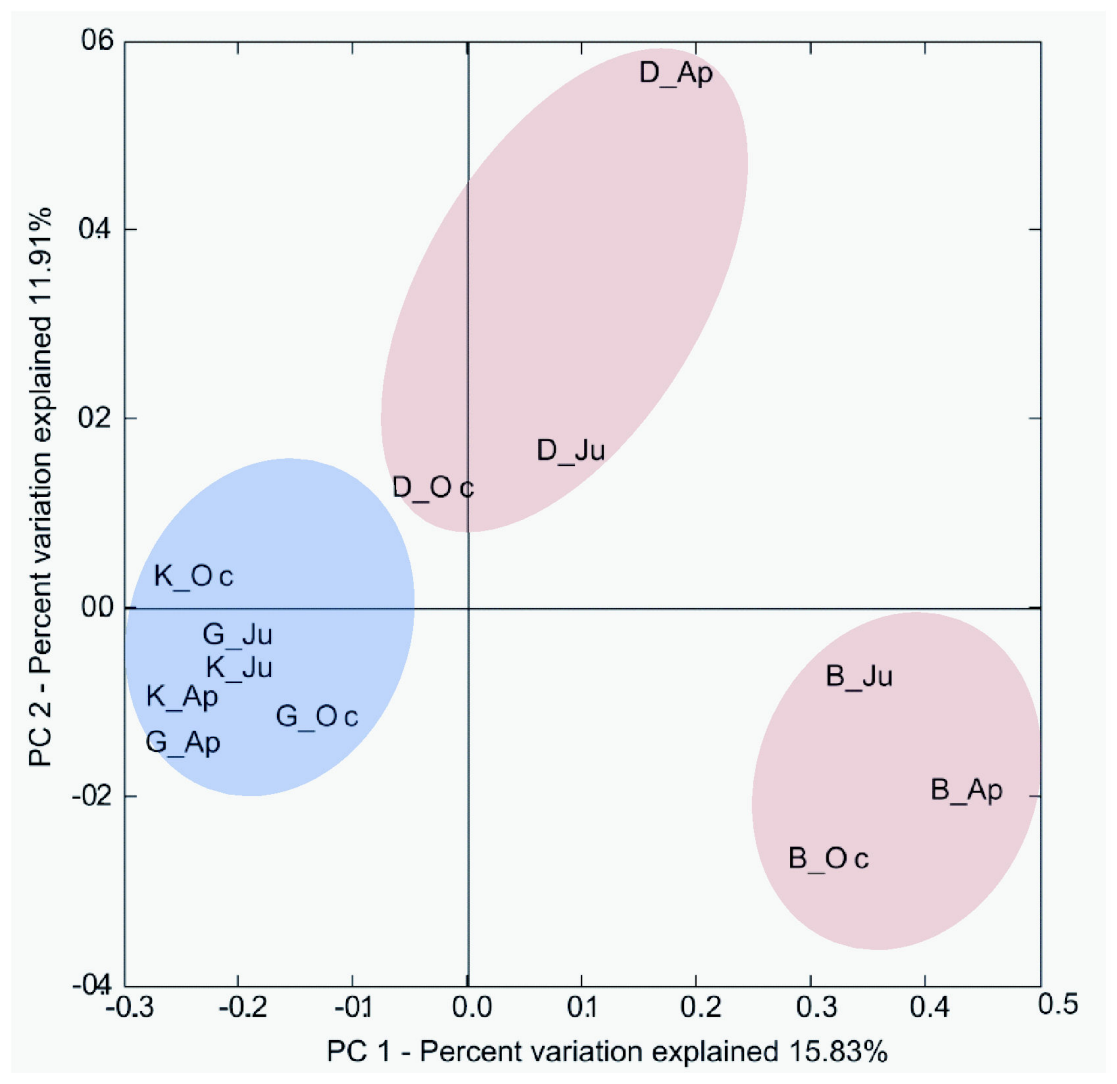
Unifrac analysis of *nif*H-gene libraries. PCoA of *nif*H-gene sequences using 1921 randomly chosen sequences per sample, from normalized weighted analysis.

Rarefaction analysis of the forward read based libraries resulted in different saturation profiles, suggesting that the *nif*H gene diversities were different across the soils (Figure S3). However, estimates of species richness are thought to be dependent on the sampling effort [51]. Therefore, the dataset was randomly resampled to the same sequencing depth (1,921 sequences per treatment) to allow for more realistic comparisons of diversity and richness among samples (Figure 3). This normalization procedure yielded total number of OTUs per sample between 40 and 79 (Table 2). These OTU numbers tended to increase with sampling time in three (B, D and K) out of the four soils (Table 2). Thus the diversity of *nif*H gene sequences, as described by the Shannon index, was slightly raised towards the end of the season. It was also higher in the clayey soils than in the sandy ones. Such sampling time and soil type effects were supported by weighted Unifrac (data not shown) and PCoA (Figure 4).

**Table 2:** Richness estimates and diversity indices for forward amplicon libraries at 90% similarity cutoff after random resampling of sequences to the same depth (1921 sequences).

**Library**	**OTUs^a^**	**Estimated OTU richness**		**Shannon^b^**
***Forward***		**Chao1**	**ACE**	
B_Ap	40	48.25 (42.16; 71.48)	53.53 (44.54; 80.31)	2.09 (2.03; 2.15)
D_Ap	61	95 (71.10; 175.41)	130.69 (107.37; 108.69)	2.79 (2.73; 2.85)
K_Ap	58	73.11 (62.76; 105.96)	123.4 (99.41; 177.03)	2.26 (2.19; 2.33)
G_Ap	58	75 (63.49; 110.55)	99.46 (82.48; 141.56)	2.60 (2.54; 2.66)
B_Ju	44	71.2 (52.63; 133.53)	59.55 (49.57; 87.45)	1.97 (1.91; 2.03)
D_Ju	52	71.43 (58.05; 114.31)	89.17 (74.59; 125.92)	2.56 (2.51; 2.61)
K_Ju	61	105.5 (75.89; 212.48)	256.19 (192.21; 375.32)	2.62 (2.55; 2.68)
G_Ju	58	85.14 (67.04; 139.51)	174.71 (142.76; 223.78)	2.54 (2.48; 2.60)
B_Oc	46	80.2 (56.82; 154.09)	134.79 (95.36; 222.26)	2.31 (2.25; 2.36)
D_Oc	79	122.5 (96.20; 188.97)	193.92 (160.06; 256.39)	2.78 (2.72; 2.85)
K_Oc	68	138.2 (92.71; 267.43)	173 (141.24; 237.01)	2.73 (2.66; 2.79)
G_Oc	49	70 (55.06; 121.68)	106.57 (88.35; 147.91)	2.53 (2.48; 2.58)

a Calculated with DOTUR at the 10% distance level.

b Shannon diversity index calculated using DOTUR (10% distance level).

Values in brackets are 95% confidence intervals as calculated using DOTUR.

**Figure 5:**
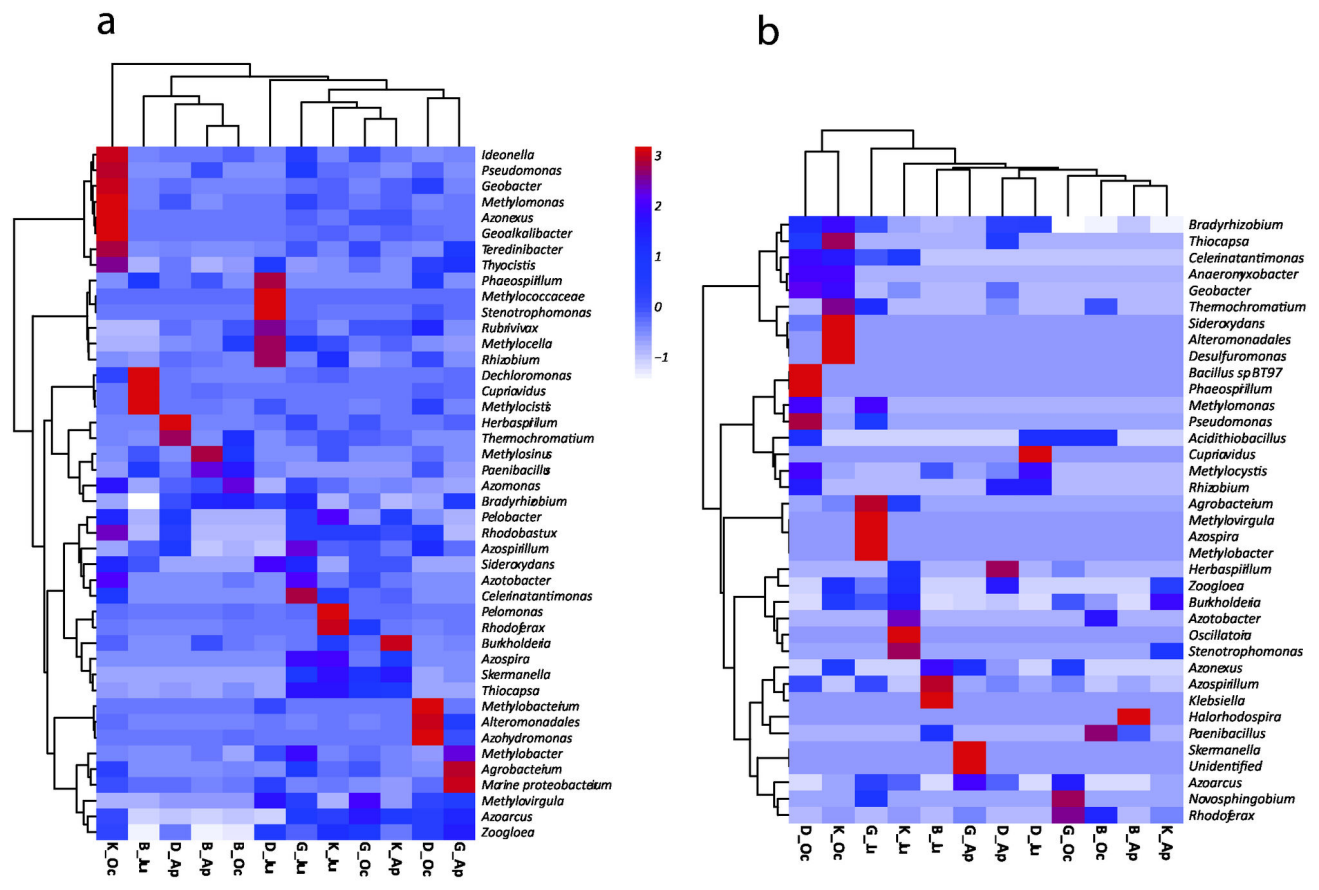
Double dendrogram and heatmap, based on the Ward minimum variance clustering method for abundant genera investigated using *nif*H gene pyrosequencing. The heatmap indicates the relative abundance of the (a) most abundant and (b) most rare genera within each sample. B, Buinen; D, Droevendaal; K, Kollumerwaard and G, Grebbedijk; Ap, April; Ju, June; Oc, October.

The compositional diversity of the *nifH* carrying communities was assessed applying a 90% similarity cut-off. This clustering level is thought to absorb most of the sequencing errors [32]. In total, the retrieved *nif*H sequences varied substantially among the soils analyzed and also over time, being distributed among Alpha-, Beta-, Gamma-, Delta- proteobacteria and Firmicutes (Figure 5). The lowest *nif*H gene diversity was observed in soil B, which was characterized mostly by the dominating *Bradyrhizobium* , *Methylocystis* *,* *Methylomonas* and *Termochromatium* species, which together made up 61% of the community. The highest diversity was observed in soil D in October (Figure 3). Soil D was dominated by species of *Azospirillum* , *Azohydromonas* , *Rhizobium* and *Herbaspirillum* , which represented 46% of the community. In soil G species of *Celerinatantimonas*, *Azoarcus* , *Burkholderia* and *Azospirillum* comprised up to 91% of the community. Soil K was dominated by *Azoarcus* , *Bradyrhizobium* , *Celerinatantimonas* and *Rhodoferax* species, making up to 71% of this soil community.

An analysis at higher taxonomic rank per soil type revealed that soils B and D were dominated by *nif*H-containing members of the class Alphaproteobacteira, around 69%, followed by 22% of Betaproteobacteria and 1% of Gammaproteobacteria. Soils K and G were dominated by a much lower percentage of Alphaproteobacteria (45%), followed by *Beta*-, with around 36%, and 17% of Gammaproteobacteria. Members of the Alphaproteobacterial classe were more than 95% similar to e.g. *Bradyrhizobium* *japonium* , *Rhizobium etli* and *Azospirillum* *canadense* . Members of Betaproteobacteria were affiliated with *Burkholderia* *vietnamiensis* , *Herbaspirillum* *seropedicae* and *Azohydromonas* *australica* , and members of the Gammaproteobacterial class were closely related to *Celerinatantimonasdiazotrophica* , *Thermochromatium* *tepidum* and *Azomonas* *agili* *.*

### Dynamics of abundant and rare nifH gene sequences

To understand which *nif*H gene types were affected by seasonality, we identified the OTUs which exhibited changes in abundance throughout the year. First, a very clear seasonal effect was observed in all soils. Interestingly, in particular cases a completely different community was found to dominate at each sampling time (Figures S4 and S5). When time was considered alone, only 15 to 37% of the OTUs were shared between sampling times. This percentage was much higher for clayey soils G (37.25%) and K (36.60%) than for sandy soils B (15%) and D (27.5%). For each soil, the stable OTUs consisted of *nif*H genes typical for *Bradyrhizobium* and *Paenibacillus* (soil B), *Azospirillum* and *Rhizobium* (soil D), *Azoarcus* and *Celerinatantimonas* (soil G) and *Celerinatantimonas* and *Burkholderia* (soil K) species. Moreover, only low percentages of OTUs were shared between soils at each sampling time, i.e. around 14-27%. In April the *nif*H gene based diversity tended to be low for all soils being represented mostly by Alphaproteobacterial members (around 55%), and specific by *Bradyrhizobium* and *Azospirillum* species. Nevertheless, diversity levels increased markedly towards the end of the season. This was very clear for soils B, D and K (Figure S4). In the June samples, similar high percentages of the alpha subdivision were found (51%), represented by *Methylomonas* , *Methylocistis*, *Azospirillum* and *Bradyrhizobium* species. An increase from 3% (in April) to 20% (in October) in abundance was observed for Gammaproteobacterial sequences, which were represented by species of *Azomonas* , *Celerinatantimonas* and *Thermochromatium* .

Some *nif*H types were spread over all the soils at most or all of the sampling times. For instance, species of the genus *Bradyrhizobium* were observed at least in two out of the three sampling times for all soils. *Azospirillum* exhibited a similar pattern. In contrast, some specific organisms could be identified as being typical for soil type and sampling time. For instance, *nif*H gene sequences typical for *Paenibacillus* *durus* , *Stenotrophomonas maltophilia* and *Dechloromonas* *aromatica* were observed only in soils B and D (sandy), whereas *Azoarcus* sp. and *Celerinatantimonasdiazotrophica* were mostly detected in clayey soils K and G (Figure 5A and Figure S4).

From the total number of clusters obtained with CD-HIT (441), 66% represented rare species (288 clusters), which made up to1.28% of the whole dataset. These rare members of the community also changed dramatically over time (Figure 5B and Figure S5). Intriguingly, most of the rare types were affiliated with Alphaproteobacteria (around 43%), followed by Beta- (33%) and Gammaproteobacteria (15%). Moreover, higher abundances of Firmicutes (3%) and Deltaproteobacterial (4%) members were found, as compared to the abundant types. Some *nif*H-types remained rare, for instance those affiliated with *Acidithiobacillus* *,* *Anaeromyxobacter* *,* *Halorhodospira* and *Novosphingobium* maintained relative abundances of approximately 0.005 (Figure 5B). Conversely, others became dominant at some point in time. For example, relative abundances of *Azonexus* increased from 0.009 to 0.067 in soil G from April to June, and those of *Zoogloea* increased from 0.007 in soil D in April to 0.97 in June (Figure 5B).

### Drivers of nifH gene-based diversity

**Figure 6:**
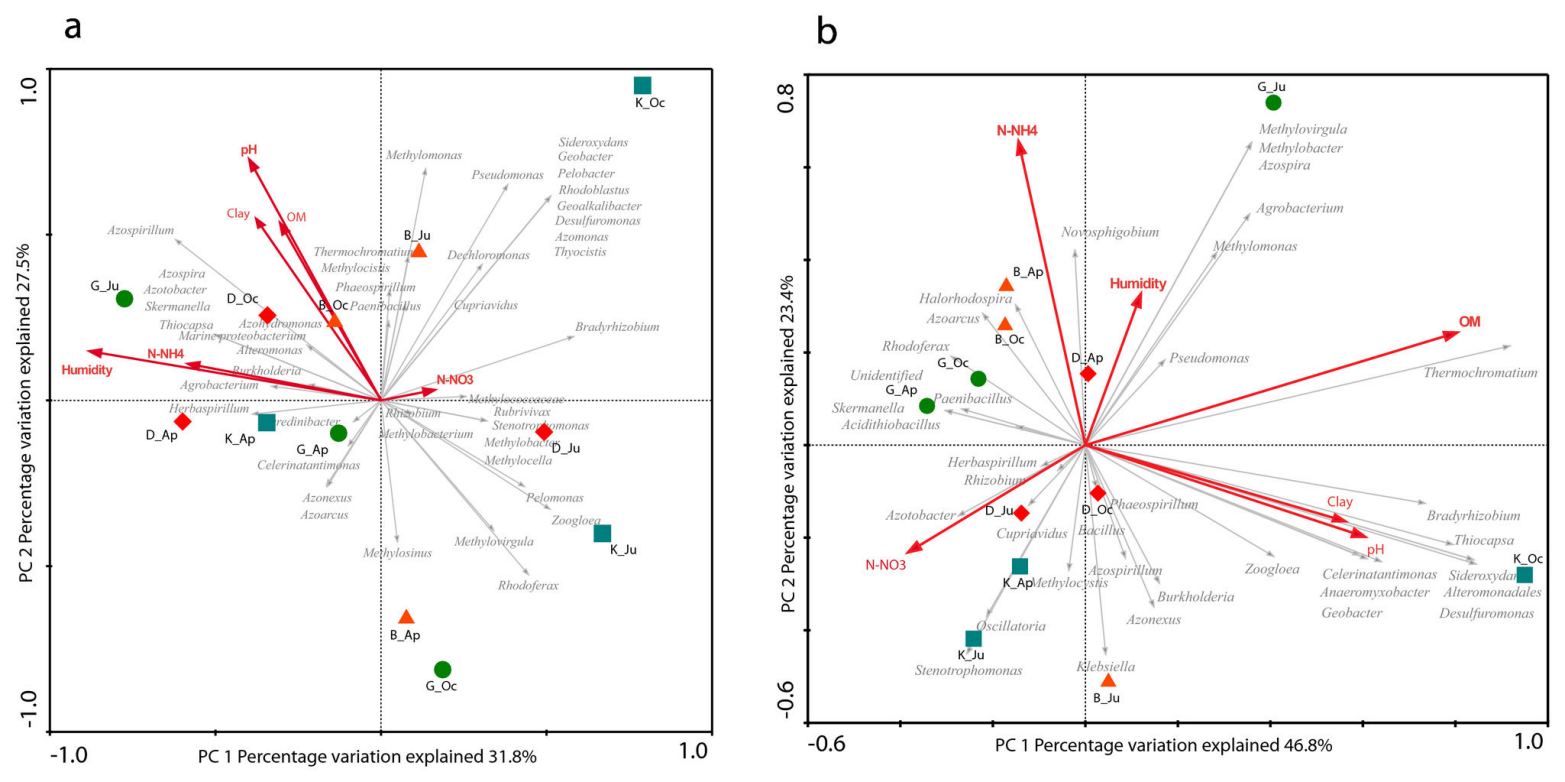
Changes in the structure of N-fixing communities from *nif*H-gene pyrosequencing across different soils and sampling times. Changes in the structure of *nif*H-gene communities and the influence of environmental parameters as revealed by RDA, considering (a) the most abundant and (b) rare sequences. The number in each axis shows the percentage of total variation explained. The length of the corresponding arrows indicated the relative importance of the geochemical factor in explaining the variation in microbial profiles. Soil samples were analyzed in four replicates at each sampling time. B, Buinen; D, Droevendaal; K, Kollumerwaard and G, Grebbedijk; Ap, April; Ju, June; Oc, October.

Forward selection on RDA analysis identified ammonium (5.0%; P = 0.032), soil pH (4.2%, P = 0.042) and clay content (4.3%; P = 0.042) as the main drivers of the *nif*H gene compositional variation of the abundant OTUs (Figure 6A). The same analysis based on the rare OTUs also identified ammonium (5.0%; P = 0.017), pH (5.1%; P = 0.009) and clay content (5.8%; P = 0.005), with the addition of nitrate (5.3%; P = 0.018) (Figure 6B).

We used Pearson’s correlation analysis to gain insights into the drivers of changes in richness, evenness and community dissimilarity (Bray, Curtis dissimilarity). Considering the abundant dataset, we observed significant direct or inverse correlations between soil pH and species richness (r = 0.728, P = 0.007), nitrate and evenness (r = -0.822, P = 0.007) and nitrate and dissimilarity (r = 0.540, P = 0.047) of N-fixing communities. Moreover, the relative abundance of *Burkholderia* species was significantly correlated to OM levels (r = 0.586, P<0.05), and those of *Azoarcus* species were driven by clay content (r = 0.747, P<0.01). Despite its ubiquity, no measured soil parameter could explain the changes in the abundance of *Bradyrhizobium* species.

## Discussion

Nitrogen fixation is carried out by N-fixing microorganisms belonging to the Archaea and Bacteria phyla. Although some of these organisms might possess nitrogenase genes, they are not necessarily able to fix N, as the *nif*H gene shares conserved regions with paralogous genes not involved in nitrogen-fixation (Clusters IV and V) [52,53]. Nevertheless, the *nif*H gene is the best-known type of nitrogenase; it is the most sequenced and has become the marker gene of choice for phylogenetic, diversity and abundance studies of nitrogen-fixing community in different environments [54]. Taking all this into account, the main idea of this work was to report the temporal and spatial fluctuations of microorganism harboring the *nif*H gene, and therefore of the microorganisms that have the potential to fix nitrogen. To do so, we made use of degenerate primers to encompass the sequence variability of the *nif*H gene [54], allowing for a greater coverage.

The population sizes of the N-fixing bacterial communities across four soils and three sampling times in the growing season were found to be within the range observed in other soil systems [47,55-57]. We observed significant seasonal fluctuations in these abundances, with a tendency to increase towards October. Temperature is known to be a key parameter that influences soil bacterial communities [58]. Moreover, along with this temperature effect, there is a variable influx of energy throughout the season. As nitrogen fixation is a energy-consuming process, it may tend to increase (in conjunction with a numerical increase of total bacterial communities) with increasing temperature and energy input, supporting the trend of a higher abundance from April (average air temperature 8°C) to June (14°C) and October (11°C). Soil nutrient status (mineral N) also changes seasonally, with generally higher levels in June, after application of fertilizers but before the intake of available nitrogen by the root system of prevailing plants [56]. This enhanced nitrogenous nutrient (ammonium and nitrate) level strongly decreased *nif*H gene abundance, as previously reported in a managed ecosystem [27], and as suggested by field evidence. For instance, free-living nitrogen fixers in the soil will preferentially use available reduced N instead of fixing it [59,60] due to the high energy demand of the latter process. However, these effects are still controversial and some may reflect other processes, such as denitrification, as some studies have reported raised *nif*H gene abundances in soils with high nitrate levels [21].

Soil pH was also identified as an important parameter controlling *nif*H gene abundances. However, soil pH might be intrinsically linked to soil type, as clayey soils tend to have higher pH than sandy soils. In a microcosm experiment in which the effects of soil pH and clay content were analyzed separately, it was observed that an increase of clay content from 60% to 80% is stressful to nitrogen fixers, which showed a drastically decreased abundance [61]. This was not observed when soil pH was raised from 4.5 to 7.5, suggesting that the higher abundances of *nif*H in clayey soils are likely due to an effect of pH rather than soil texture. Moreover, an effect of soil pH on the abundance of diazotrophs has previously been observed [4]; it co-varied with season, with stronger correlations at the beginning than at the end of the season.

Seasonal shifts were also observed in the *nif*H gene based community structure, as previously reported [4]. In fact, we found -- through *nif*H gene-based PCR-DGGE analyses--that on average, around 60% of the diazotrophic communities shifted from April to October. This stands in contrast to studies based on PCR-DGGE which have shown substantial stability of the diazotrophic assemblage over an annual cycle [62,63]. In conjunction with a previous analysis of the same communities at a different sampling time [4], our results indicate an internal restructuring of the diazotrophic community. This was apparently not stochastic, and it occurred to a greater extent in the sandy soils than in the clayey ones. Moreover, variation partitioning showed that nitrate, ammonium and clay content were related to the structural changes. The effects of N availability on the diazotrophic community structures are as-yet not clear. Although some studies reported no correlation between community structure and N fertilization [63,64], we found a significant percentage of the community variation being explained by nitrate (28.6%) and ammonium levels (16.1%), which is in accordance with the physiology of nitrogen fixation (common response to N availability). Besides N, soil texture also represents a critical variable, as clay fractions in soils are important in forming micro aggregates [10]. Up to 70% of the free-living nitrogen-fixing bacteria are often found in soil clay fractions [65]. Occurrence in soil clay fractions is possibly favorable, as microaerophilic or anaerobic conditions prevail in such sites, providing more adequate environments for the oxygen-sensitive nitrogen fixation process.

In order to identify and characterize the members of the N-fixing communities in our samples, we developed a pyrosequencing approach based on the *nif*H gene. In a recent study, several primer sets designed to amplify *nif*H gene--including the ones used in this study--were explored in an *in silico* analysis of specificity and coverage was performed [54]. Here, we observed that the primers used in this study for the pyrosequencing of *nif*H gene did not amplify alternative *nif*- genes, indicating that they are suitable for diversity studies of potential nitrogen-fixing microorganisms. Overall, our results confirmed the prediction that the nitrogen-fixing microorganisms would be highly diverse [47,66] and dynamic [4], consistent with the results from the PCR-DGGE analyses. The diversity and evenness values were strongly driven by local soil conditions. Furthermore, the evenness and dissimilarities of the communities were affected by nitrate levels, which led to very unequal communities. In a study of the effect of N inputs on soil bacterial communities, it was observed that as N inputs increased, communities became more distinct from those not receiving N fertilizer, and the response of bacterial diversity was inconsistent [67]. Furthermore, a clear influence of soil type and pH on the composition of the N-fixing communities was indicated by UniFrac analysis, confirming the results obtained by CCA analysis of the PCR-DGGE patterns. In this sense we observed that, whereas the presence or absence of different types was driven by soil pH or texture [61], other factors such as nitrate and ammonium drive the differential abundance of these types in each treatment (seen from weighted Unifrac).

Our pyrosequencing dataset revealed strong seasonal variations in the composition of the *nif*H gene community sequences, confirming previous results from PCR-DGGE data [4]. Temporal variability in the N fixers is often reported in marine environments [68-70], but it has been rarely studied in terrestrial ecosystems [4,47]. The sequences recovered were affiliated with several major groups of bacteria, which changed with sampling time. The sandy soils B and D showed the lowest percentage of shared species between sampling times and hence large fractions of the nitrogen-fixing communities in such soils may be under constant replacement, with some populations disappearing and reappearing during the time period analyzed. Similar results have recently been provided by Gobet et al. [70] who studied the effects of seasonality and sediment depth on the fluctuations of total bacterial communities of marine sands. In this study, when time was considered alone, only 18-37% of unique OTUs were shared between any two sampling times. In our data set, however, some types revealed clear trends in their seasonal dynamics. For instance, *nifH* genes allocated in the class Gammaproteobacteria (mostly *Methyloccocales*, *Pseudomonadales* and *Chromatiales*) increased significantly in the course of the season. This increase could be explained by an increase in soil nutrients in June and October when compared to April, due to the presence of plants and root exudation. As previously suggested, this response might reflect the copiotrophic behavior of members of this group, which tend to be favored under nutrient-rich conditions [71,72].

Cosmopolitan *nif*H species were identified as species that were present across time and soil type. In this class, we invariably found *Bradyrhizobium* , *Azospirilum* and *Methylocystis* to be in high abundance, which is consistent with data presented earlier [4,73,74]. The dominance of *nif*H gene types affiliated with *Alphaproteobacteria* across all soils and sampling times was consistent with the presumed dominance of this group, observed for terrestrial [75], and marine environments [70]. The high abundance of sequences affiliated with *Bradyrhizobium* is noteworthy, as it is known as a symbiotic N-fixer. In addition, there is an increasing understanding that this organism may also play key metabolic role as a soil saprophyte, being able to colonize soil and rhizosphere in the presence or absence of plants [76], under which condition they may even denitrify [77]. The fields we investigated are used for potato cropping and were subjected to crop rotations including non-leguminous plants. It is highly likely that the *Bradyrhizobium* types found are excellent survivors across the diverse conditions applied in these farming systems. *Azospirillum* , on the other hand, is a genus encompassing non-symbiotic (yet plant-associated) N-fixers known to be associated with roots of grasses, cereals, food crops and soils [78]. Our results corroborate previous evidence based on cultivation techniques which highlight the cosmopolitan distribution of this diazotrophs [79,80].

We also detected particular (“core”) species in each soil, that is, species that were observed only in a particular soil, soil type and/or at a particular sampling time. Such core species could represent types that are unique to a particular soil or condition, but are sensitive to environmental variations (e.g. *Paenibacillus* , *Burkholderia* and *Celerinatantimonas* species). The low percentage of shared OTUs among the soils suggested that each soil, with its own structure and characteristics, harbored a particular N-fixing community. Indeed, in previous work, soil type has been reported to be the primary driver of soil bacterial composition [81]. Here, we posit that the community make-up of soil N fixers is determined by the (long) history of the soil under study. The community will consist of free-living and plant-associated nitrogen fixers, as niches for such functional groups will commonly exist in soils. In line with our data, fluctuations in the communities occur regularly, and groups that dominate at one point in time may have dwindled away in a next time point. Overall, however, there will be selection for the function at times, i.e. when reduced nitrogen becomes limiting for the soil biota. This incidental selection for function will tend to favor organisms that thrive under temporally defined conditions of temperature, pH and OM levels, and hence we may at the basis of the strongly differing N-fixing communities observe such differential selections for the very same function. In other words, function is maintained in the community, phylogeny of the underlying players is not.

Thus our results indicate an interaction between sampling time and environment with respect to effects on the evenness of the community, causing an increase in abundance of dominant OTUs in detriment of others at the beginning of the season. In the present study, three main environmental variables were identified as main drivers of the nitrifying communities, i.e ammonium, pH and clay content. Indeed, although the *nif*H gene based diversity was high in the clayey soils, a lower turnover rate through time was found. An effect of ammonium on nitrogen fixation has been recognized for a long time, as nitrogenase is commonly inhibited by the presence of NH_4_^+^ ions [82,83]. The abundant *nif*H-carrying bacterial groups are thought to be well-adapted to their environment [84]; however, rare diazotrophic species also responded to the same environmental parameters, indicating their functional importance. Thus the physicochemical properties of the soils might select both the dominant and the rare types, corroborating Hsu and Buckley [3]. Notably, some rare members increased their abundance at some point in time, when appropriate conditions were met, thus acting as source populations.

This is the first in-depth sequencing analysis of the *nif*H gene in agricultural soils along a temporal scale (one growing season), providing a wealth of new data and insights into the extent diazotrophic communities. The dominant and rare *nif*H gene types showed great spatial and temporal variations, mainly due to different N availabilities and soil pH levels. While some *nif*H gene types were always present (e.g. *Bradyrhizobium* ), others only became apparent when conditions presumably provided an adequate environment (e.g. *Paenibacillus* ). Furthermore, the broad data set obtained in this study provides a basis for directed studies on individual performance, e.g. of the cosmopolitan versus specific *nif*H containing species.

### Ethic statement

No specific permits were required for the described field studies. The locations are not protected. The field studies did not involve endangered or protected species.

## Supporting Information

Figure S1 **Richness of bacterial and N-fixing communities estimated as the number of DGGE bands.** B, Buinen; D, Droevendaal; K, Kollumerwaard and G, Grebbedijk; Ap, April; Ju, June; Oc, October.
(EPS)

Figure S2 **Unweighted pair group method with average linkages dendograms of diazotrophic community associated with four soils obtained from analysis of DGGE fingerprints.**
(EPS)

Figure S3 **Rarefaction analysis of the diversities of *nif*H gene in the four soils and three sampling.** The OTUs were classified at 90% similarity cutoff based on amino acid sequences. B, Buinen; D, Droevendaal; K, Kollumerwaard and G, Grebbedijk; Ap, April; Ju, June; Oc, October.
(EPS)

Figure S4 **Taxonomic classification of the most abundant (“core”) *nif*H-gene sequences associated with the two sandy agricultural soils (B and D) at three sampling times, April, June and October.** Multi-colored charts at the legend are shown for each sample correspondingly.
(PNG)

Figure S5 **Taxonomic classification of the less abundant (“rare”) *nif*H-gene sequences associated with the two clayey agricultural soils (G and K) at three sampling times, April, June and October.** Multi-colored charts at the legend are shown for each sample correspondingly.
(PNG)

Table S1 **Soil characteristics measured in this study.**
(DOCX)

Table S2 **PCR and cycling conditions for PCR-DGGE analysis and real time quantification of *nif*H gene.**
(DOCX)

Table S3 **PCR and cycling conditions for pyrosequencing of *nif*H gene.**
(DOCX)

Table S4 **Forward and reverse barcoded primers used for pyrosequencing of *nif*H gene.**
(DOCX)

Table S5 **Richness estimates and diversity indices for forward and reverse amplicon libraries at 90% similarity cutoff.**
(DOCX)

## References

[1] Capone DG, Knapp AN (2007) Oceanography: a marine nitrogen cycle fixation. Nature 445: 159-160. doi:10.1038/445159a. PubMed: 17215835. 17215835 10.1038/445159a

[2] Dixon R, Kahn D (2004) Genetic regulation of biological nitrogen fixation. Nat Rev Microbiol 2: 621-631. doi:10.1038/nrmicro954. PubMed: 15263897. 15263897 10.1038/nrmicro954

[3] Hsu SF, Buckley DH (2009) Evidence for the functional significance of diazotroph community structure in soil. ISME J 3: 124-136. doi:10.1038/ismej.2008.82. PubMed: 18769458. 18769458 10.1038/ismej.2008.82

[4] Pereira e Silva MC, Semenov AV, van Elsas JD, Salles JF (2011) Seasonal variation in diversity and abundance of diazotrophic communities across soils. FEMS Microbiol Ecol 77: 57-68. doi:10.1111/j.1574-6941.2011.01081.x. PubMed: 21385188. 21385188 10.1111/j.1574-6941.2011.01081.x

[5] Peoples MB, Herridge DF, Ladha JK (1995) Biological nitrogen fixation: an efficient source of nitrogen for sustainable agricultural production? Plant Soil 174: 3-28. doi:10.1007/BF00032239.

[6] Reed SC, Cleveland CC, Townsend AR (2011) Functional ecology of free-living nitrogen fixation: A contemporary perspective. Annu Rev Ecol Evol Syst 42: 489–512. doi:10.1146/annurev-ecolsys-102710-145034.

[7] Bürgmann H, Widmer F, Von Sigler W, Zeyer J (2004) New molecular screening tools for analysis of free-living diazotrophs in soil. Appl Environ Microbiol 70: 240–247. doi:10.1128/AEM.70.1.240-247.2004. PubMed: 14711647. 14711647 10.1128/AEM.70.1.240-247.2004 PMC321232

[8] Roper M, Smith N (1991) Straw decomposition and nitrogenase activity (C_2_H_2_ reduction) by free-living microorganisms from soil: Effects of pH and clay content. Soil Biol Biochem 23: 275-283. doi:10.1016/0038-0717(91)90064-Q.

[9] Reed SC, Cleveland CC, Townsend AR (2008) Tree species control rates of free-living nitrogen fixation in a tropical rain forest. Ecology 89: 2924-2934. doi:10.1890/07-1430.1. PubMed: 18959329. 18959329 10.1890/07-1430.1

[10] Gupta VVSR, Roper MM, Roget DK (2006) Potential for non-symbiotic N2- fixation in different agroecological zones of southern Australia. Aust J Soil Res 44: 343–354. doi:10.1071/SR05122.

[11] Schloter M (2003) Indicators for evaluating soil quality. Agric Ecosyst Environ 98: 255-262. doi:10.1016/S0167-8809(03)00085-9.

[12] Ollivier J, Töwe S, Bannert A, Hai B, Kastl E-M et al. (2011) Nitrogen turnover in soil and global change. FEMS Microbiol Ecol 78: 3-16. doi:10.1111/j.1574-6941.2011.01165.x. PubMed: 21707675. 21707675 10.1111/j.1574-6941.2011.01165.x

[13] Pankhurst CE, Hawke BG, McDonald HJ, Kirkby CA, Buckerfield JC et al. (1995) Evaluation of soil biological properties as potential bioindicators of soil health. Aust J Experim Agric 35: 1018-1028.

[14] Doran JW, Safley M (1997) Defining and assessing soil health and sustainable productivity. In: Pankhurst CE Doube BM Gupta VSR Biological Indicators of Soil Quality.

[15] Hooper DU, Vitousek PM (1997) The effects of plant composition and diversity on ecosystem processes. Science 277: 1302–1305. doi:10.1126/science.277.5330.1302.

[16] Tilman D, Knops J, Wedin D, Reich P, Ritchie M et al. (1997) The influence of functional diversity and composition on ecosystem processes. Science 277: 1300–1302. doi:10.1126/science.277.5330.1300.

[17] Reed SC, Cleveland CC, Towsend AR (2007) Controls over leaf litter and soil nitrogen fixation in lowland tropical rain forests. Biotropica 39: 585-592. doi:10.1111/j.1744-7429.2007.00310.x.

[18] Pérez CA, Carmona MR, Aravena JC, Armesto JJ (2004) Successional changes in soil nitrogen availability, nonsymbiotic nitrogen fixation and carbon/nitrogen ratios in southern Chilean forest ecosystems. Oecologia 140: 617–625. PubMed: 15221437. 15221437 10.1007/s00442-004-1627-y

[19] Steward KJ, Coxson D, Siciliano SD (2011) Small-scale spatial patterns in N_2_-fixation and nutrient availability in arctic hummock-hollow ecosystem. Soil Biol Biochem 43: 133-140. doi:10.1016/j.soilbio.2010.09.023.

[20] Bentley BL (1987) Nitrogen fixation by epiphylls in a tropical rain forest. Ann Mo Bot Gard 74: 234–241. doi:10.2307/2399396.

[21] Mergel A, Kloos K, Bothe H (2001) Seasonal fluctuations in the population of denitrifying and N2 -fixing bacteria in an acid soil of a Norway spruce forest. Plant Soil 230: 145-160. doi:10.1023/A:1004826116981.

[22] Patra AK, Abbadie L, Clays-josserand A, Degrange V, Grayston SJ et al. (2006) Effects of management regime and plant species on the enzyme activity and genetic structure of N-fixing, denitrifying and nitrifying bacterial communities in grassland soils. Environ Microbiol 8: 1005-1016. doi:10.1111/j.1462-2920.2006.00992.x. PubMed: 16689721. 16689721 10.1111/j.1462-2920.2006.00992.x

[23] Salles JF, van Elsas JD, van Veen JA (2006) Effect of agricultural management regime on Burkholderia community structure in soil. Microb Ecol 52: 267-279. doi:10.1007/s00248-006-9048-6. PubMed: 16897309. 16897309 10.1007/s00248-006-9048-6

[24] Wakelin SA, Gregg AL, Simpson RJ, Li GD, Riley IT et al. (2009) Pasture management clearly affects soil microbial community structure and N-cycling bacteria. Pedobiologia 52: 237-251. doi:10.1016/j.pedobi.2008.10.001.

[25] Poly F, Monrozier LJ, Bally R (2001b) Improvement in the RFLP procedure for studying the diversity of nifH genes in communities of nitrogen fixers in soil. Res Microbiol 52: 95-103. PubMed: 11281330. 10.1016/s0923-2508(00)01172-4 11281330

[26] Wakelin SA, Colloff MJ, Harvey PR, Marschner P, Gregg AL et al. (2007) The effects of stubble retention and nitrogen application on soil microbial community structure and functional gene abundance under irrigated maize. FEMS Microbiol Ecol 59: 661–670. doi:10.1111/j.1574-6941.2006.00235.x. PubMed: 17116166. 17116166 10.1111/j.1574-6941.2006.00235.x

[27] Lindsay EA, Colloff MJ, Gibb NL, Wakelin SA (2010) The abundance of microbial functional genes in grassy woodlands is influenced more by soil nutrient enrichment than by recent weed invasion or livestock exclusion. Appl Environ Microbiol 76: 5547–5555. doi:10.1128/AEM.03054-09. PubMed: 20601513. 20601513 10.1128/AEM.03054-09 PMC2918952

[28] Orchard ED, Webb EA, Dyhrman ST (2009) Molecular analysis of the phosphorus starvation response in *Trichodesmium* spp. Environ Microbiol 11: 2400–2411. doi:10.1111/j.1462-2920.2009.01968.x. PubMed: 19555381. 19555381 10.1111/j.1462-2920.2009.01968.x

[29] van Elsas JD, Duarte GF, Keijzer-Wolters A, Smit E (2000) Analysis of the dynamics of fungal communities in soil via fungal-specific PCR of soil DNA followed by denaturing gradient gel electrophoresis. J Microbiol Methods 43: 133-151. doi:10.1016/S0167-7012(00)00212-8. PubMed: 11121612. 11121612 10.1016/s0167-7012(00)00212-8

[30] Hartmann M, Widmer F (2006) Community structure analyses are more sensitive to differences in soil bacterial communities than anonymous diversity indices. Appl Environ Microbiol 72: 7804-7812. doi:10.1128/AEM.01464-06. PubMed: 17041161. 17041161 10.1128/AEM.01464-06 PMC1694274

[31] Quince C, Lanzén A, Curtis TP, Davenport RJ, Hall N et al. (2009) Accurate determination of microbial diversity from 454 pyrosequencing data. Nat Methods 9: 639-641. PubMed: 19668203. 10.1038/nmeth.1361 19668203

[32] Kunin V, Engelbrektson A, Ochman H, Hugenholtz P (2009) Wrinkles in the rare biosphere: pyrosequencing errors lead to artificial inflation of diversity estimates. Environ Microbiol 12: 118–123. PubMed: 19725865. 19725865 10.1111/j.1462-2920.2009.02051.x

[33] Pereira e Silva MC, Poly F, Guillarmaud N, van Elsas JD, Salles JF (2012) Fluctuations in ammonia oxidizer communities are driven by soil structure and pH Front Microbiol. p. 3:77 doi:10.3389/fmicb.2012.00077. 10.3389/fmicb.2012.00077 PMC3291871 22403578

[34] Töwe S, Albert A, Kleineidam K, Brankatschk R, Dümig A et al. (2010) Abundance of microbes involved in nitrogen transformation in the rhizosphere of *Leucanthemopsis* *alpina* (L.) Heywood grown in soils from different sites of the Damma Glacier Forefield. Microb Ecol 60: 762-770. doi:10.1007/s00248-010-9695-5. PubMed: 20549199. 20549199 10.1007/s00248-010-9695-5

[35] Gomes NCM, Heuer H, Schonfeld J, Costa R, Hagler-Mendonça L et al. (2001) Bacterial diversity of the rhizosphere of maize (Zea mays) grown in tropical soil studied by temperature gradient gel electrophoresis. Plant Soil 232: 167-180. doi:10.1023/A:1010350406708.

[36] Brons JK, van Elsas JD (2008) Analysis of bacterial communities in soil by use of denaturing gradient gel electrophoresis and clone libraries, as influenced by different reverse primers. Appl Environ Microbiol 74: 2717-2727. doi:10.1128/AEM.02195-07. PubMed: 18310425. 18310425 10.1128/AEM.02195-07 PMC2394888

[37] Diallo MD, Willems A, Vloemans N, Cousin S, Vandekerckhove TT, Lajudie P, Neyra M et al. (2004) Polymerase chain reaction denaturing gradient gel electrophoresis analysis of the N2-fixing bacterial diversity in soil under *Acacia* *tortilis* ssp. *raddiana* and *Balanites* *aegyptiaca* in the dryland part of Senegal. Environ Microbiol 6: 400-415. doi:10.1111/j.1462-2920.2004.00577.x. PubMed: 15008817. 15008817 10.1111/j.1462-2920.2004.00577.x

[38] Simonet P, Grosjean MC, Arvind KM, Nazaret S, Cournoyer B, Normand P (1991) Frankia genus-specific characterization by polymerase chain reaction. Appl Environ Microbiol 57: 3278-3286. PubMed: 1781685. 1781685 10.1128/aem.57.11.3278-3286.1991 PMC183960

[39] Poly F, Ranjard L, Goubiére SNF, Monrozier LJ (2001a) Comparison of nifH Gene Pools in Soils and Soil Microenvironments with Contrasting Properties. Appl Environ Microbiol 67: 2255-2262. doi:10.1128/AEM.67.5.2255-2262.2001. PubMed: 11319109. 11319109 10.1128/AEM.67.5.2255-2262.2001 PMC92864

[40] Bach H-J, Tomanova J, Schloter M, Munch JC (2002) Enumeration of total bacteria and bacteria with genes for proteolytic activity in pure cultures and in environmental samples by quantitative PCR mediated amplification. J Microbiol Met 49: 235-245. doi:10.1016/S0167-7012(01)00370-0. PubMed: 11869788. 10.1016/s0167-7012(01)00370-0 11869788

[41] Ritz C, Spiess AN (2008) qPCR: an R package for sigmoidal model selection in quantitative real-time polymerase chain reaction analysis’. Bioinformatics 24: 1549-1551. doi:10.1093/bioinformatics/btn227. PubMed: 18482995. 18482995 10.1093/bioinformatics/btn227

[42] Roesch LFW, Olivares FL, Passaglia LMP, Selbach PA, Saccol de Sa EL et al. (2006) Characterization of diazotrophic bacteria associated with maize: effect of plant genotype, ontogeny and nitrogen-supply. World J Microbiol Biotechnol 22: 967-974. doi:10.1007/s11274-006-9142-4.

[43] Huse SM, Huber JA, Morrison HG, Sogin ML, Welch DM (2007) Accuracy and quality of massively parallel DNA pyrosequencing. Genome Biol 8: R143. doi:10.1186/gb-2007-8-7-r143. PubMed: 17659080. 17659080 10.1186/gb-2007-8-7-r143 PMC2323236

[44] Gouy M, Guindon S, Gascuel O (2010) SeaView version 4 : a multiplatform graphical user interface for sequence alignment and phylogenetic tree building. Mol Biol Evol 27: 221-224. doi:10.1093/molbev/msp259. PubMed: 19854763. 19854763 10.1093/molbev/msp259

[45] Schloss PD, Handelsman J (2008) A statistical toolbox for metagenomics: assessing functional diversity in microbial communities. BMC Bioinformatics 15: 1-15. 10.1186/1471-2105-9-34 PMC2238731 18215273

[46] Palmer K, Drake HL, Horn MA (2009) Genome-derived criteria for assigning environmental narG and nosZ sequences to operational taxonomic units of nitrate reducers. Appl Environ Microbiol 75: 5170–5174. doi:10.1128/AEM.00254-09. PubMed: 19502444. 19502444 10.1128/AEM.00254-09 PMC2725509

[47] Mao Y, Yannarell AC, Mackie RI (2011) Changes in N-transforming Archaea and Bacteria in soil during the establishment of bioenergy crops. PLOS ONE 6(9): e24750. doi:10.1371/journal.pone.0024750. PubMed: 21935454. 21935454 10.1371/journal.pone.0024750 PMC3173469

[48] Li W, Godzik A (2006) Cd-hit: a fast program for clustering and comparing large sets of protein or nucleotide sequences. Bioinformatics 22: 1658-1659. doi:10.1093/bioinformatics/btl158. PubMed: 16731699. 16731699 10.1093/bioinformatics/btl158

[49] Tamura K, Peterson D, Peterson N, Stecher G, Nei M et al. (2011) Mega5: Molecular evolutionary genetics analysis using maximum likelihood, evolutionary distance and maximum parsimony methods. Mol Biol Evol 28: 2731-2739. doi:10.1093/molbev/msr121. PubMed: 21546353. 21546353 10.1093/molbev/msr121 PMC3203626

[50] Lozupone C, Hamady M, Knight R (2006) UniFrac – An online tool for comparing microbial community diversity in a phylogenetic context. BMC Bioinformatics 14: 1-14. 10.1186/1471-2105-7-371 PMC1564154 16893466

[51] Hughes JB, Hellmann JJ, Ricketts TH, Bohannan BJM (2001) Counting the uncountable: statistical approaches to estimating microbial diversity. Appl Environ Microbiol 67: 4399-4406. doi:10.1128/AEM.67.10.4399-4406.2001. PubMed: 11571135. 11571135 10.1128/AEM.67.10.4399-4406.2001 PMC93182

[52] Raymond J, Siefert JL, Staples CR, Blankenship RE (2004) The natural history of nitrogen fixation. Mol Biol Evol 21: 541-554. PubMed: 14694078. 14694078 10.1093/molbev/msh047

[53] Schlessman JL, Woo D, Joshua-Tor L, Howard JB, Rees DC (1998) Conformational variability in structures of the nitrogenase iron protein from Azotobacter vinelandii and Clostridium pasteurianum. J Mol Biol 280: 669-685. doi:10.1006/jmbi.1998.1898. PubMed: 9677296. 9677296 10.1006/jmbi.1998.1898

[54] Gaby JC, Buckley DH (2012) A comprehensive evaluation of PCR primers to amplify the *nif*H gene of nitrogenase. PLOS ONE 7(7): e42149. doi:10.1371/journal.pone.0042149. PubMed: 22848735. 22848735 10.1371/journal.pone.0042149 PMC3405036

[55] Hai B, Diallo NH, Sall S, Haesler F, Schauss K et al. (2009) Quantification of key genes steering the microbial nitrogen cycle in the rhizosphere of sorghum cultivars in tropical agroecosystems. Appl Environ Microbiol 75: 4993–5000. doi:10.1128/AEM.02917-08. PubMed: 19502431. 19502431 10.1128/AEM.02917-08 PMC2725491

[56] Orr CH, James A, Leifert C, Cooper JM, Cummings SP (2010) Diversity and activity of free-living nitrogen-fixing bacteria and total bacteria in organic and conventionally managed soils. Appl Environ Microbiol 77: 911-917. PubMed: 21131514. 21131514 10.1128/AEM.01250-10 PMC3028727

[57] Huang L-N, Tang F-Z, Song Y-S, Wan C-Y, Wang S-L et al. (2011) Biodiversity, abundance, and activity of nitrogen-fixing bacteria during primary succession on a copper mine tailings. FEMS Microbiol Ecol 78: 439-450. doi:10.1111/j.1574-6941.2011.01178.x. PubMed: 22066852. 22066852 10.1111/j.1574-6941.2011.01178.x

[58] Pettersson M, Bååth E (2003) Temperature-dependent changes in the soil bacterial community in limed and unlimed soil. FEMS Microbiol Ecol 45: 13-21. doi:10.1016/S0168-6496(03)00106-5. PubMed: 19719602. 19719602 10.1016/S0168-6496(03)00106-5

[59] Barron AR, Wurzburger N, Bellenger JP, Wright SJ, Kraepiel AML et al. (2008) Molybdenum limitation of asymbiotic nitrogen fixation in tropical forest soils. Nat Geosci 2: 42–45.

[60] Cusack DF, Silver W, McDowell WH (2009) Biological nitrogen fixation in two tropical forests: ecosystem level patterns and effects of nitrogen fertilization. Ecosystems 12: 1299–1315. doi:10.1007/s10021-009-9290-0.

[61] Pereira e Silva MC (2013) The normal operating range of soil functioning: understanding the natural fluctuations of N cycling communities. Thesis http://dissertations.ub.rug.nl/faculties/science/2013/m.de.cassia.pereira/.

[62] Piceno YM, Noble PA, Lovell CR (1999) Spatial and temporal assessment of diazotroph assemblage composition in vegetated salt marsh sediments using denaturing gradient gel electrophoresis analysis. Microb Ecol 38: 157-167. doi:10.1007/s002489900164. PubMed: 10441708. 10441708 10.1007/s002489900164

[63] Piceno YM, Lovell CR (2000) Stability in natural bacterial communities: I. Nutrient addition effects on rhizosphere diazotrophic assemblage composition. Microb Ecol 39: 32-40. doi:10.1007/s002489900192. PubMed: 10790515. 10790515 10.1007/s002489900192

[64] Schäfer H, Bernard L, Courties C, Lebaron P, Servais P et al. (2001) Microbial community dynamics in Mediterranean nutrient-enriched seawater mesocosms: changes in the genetic diversity of bacterial populations. FEMS Microbiol Ecol 34: 243–253. doi:10.1111/j.1574-6941.2001.tb00775.x. PubMed: 11137604. 11137604 10.1111/j.1574-6941.2001.tb00775.x

[65] Chotte J, Shwartzmann A, Bally R, Monrozier JL (2002) Changes in bacterial communities and Azospirillum diversity in soil fractions of a tropical soil under 3 or 19 years of natural fallow. Soil Biol Biochem 34: 1083-1092. doi:10.1016/S0038-0717(02)00041-X.

[66] Duc L, Noll M, Meier BE (2009) High diversity of diazotrophs in the forefield of a receding alpine glacier. Microbiol Ecol 57: 179-190. doi:10.1007/s00248-008-9408-5. 10.1007/s00248-008-9408-5 18563478

[67] Ramirez KS, Craine JM, Fierer N (2010) Nitrogen fertilization inhibits soil microbial respiration regardless of the form of nitrogen applied. Soil Biol Biochem 12: 2336–2338.

[68] Foster RA, Paytan A, Zehr JP (2009) Seasonality of N2 fixation and nifH gene diversity in the Gulf of Aqaba (Red Sea). Limnol Oceanogr 59: 219-233.

[69] Kong L, Jing H, Kataoka T, Sun J, Liu H (2011) Phylogenetic diversity and spatio-temporal distribution of nitrogenase genes (nifH) in the northern South China Sea. Aquat Microb Ecol 65: 15-27. doi:10.3354/ame01531.

[70] Gobet A, Böer SI, Huse SM, van Beusekom JEE, Quince C et al. (2012) Diversity and dynamics of rare and of resident bacterial populations in coastal sands. ISME J 6: 542-553. doi:10.1038/ismej.2011.132. PubMed: 21975598. 21975598 10.1038/ismej.2011.132 PMC3280144

[71] Zavarzin GA, Stackebrandt E, Murray RGE (1991) A correlation of phylogenetic diversity in the Proteobacteria with the influences of ecological forces. Can J Microbiol 37: 1-6. doi:10.1139/m91-001. PubMed: 1708691. 1708691 10.1139/m91-001

[72] Fierer N, Lauber CL, Ramirez KS, Zaneveld J, Bradford MA et al. (2012) Comparative metagenomic, pylogenetic and physiological analyses of soil microbial communities across nitrogen gradients. ISME J 6: 1007-1017. doi:10.1038/ismej.2011.159. PubMed: 22134642. 22134642 10.1038/ismej.2011.159 PMC3329107

[73] Chowdhury SP, Schmid M, Hartmann A, Tripathia AK (2009) Diversity of 16S-rRNA and nifH genes derived from rhizosphere soil and roots of an endemic rough tolerant grass, *Lasiurus* *sindicus* . Eur J Soil Biol 45: 114-122. doi:10.1016/j.ejsobi.2008.06.005.

[74] Coelho MRR, Marriel IE, Jenkins SN, Lanyon CV, Seldin L et al. (2008) Molecular detection and quantification of nifH gene sequences in the rhizosphere of sorghum (*Sorghum* *bicolor*) sown with two levels of nitrogen fertilizer. Appl Soil Ecol 42: 48–53.

[75] Smit E, Leeflang P, Gommans S, van den Broek J, van Mil S et al. (2001) Diversity and seasonal fluctuations of the dominant members of the bacterial soil community in a wheat field as determined by cultivation and molecular methods. Appl Environ Microbiol 67: 2284-2291. doi:10.1128/AEM.67.5.2284-2291.2001. PubMed: 11319113. 11319113 10.1128/AEM.67.5.2284-2291.2001 PMC92868

[76] Chatel DL, Greenwood RM, Parker CA (1968) Saprophytic competence as an important character in the selection of Rhizobium for inoculation. In Trans. 9th Int. Cong. Soil Sci Adelaide: 11–65. Int. Soc. Soil Sci. Publisher, Sydney.

[77] O’Hara GW, Daniel RM (1985) Rhizobial denitrification: A review. Soil Biol Biochem 1: 1–9.

[78] Peng G, Wang H, Zhang G, Hou W, Liu Y et al. (2006) *Azospirillum* *melinis* sp. nov., a group of diazotrophs isolated from tropical molasses grass. Int J Syst Evol Microbiol 56: 1263–1271. doi:10.1099/ijs.0.64025-0. PubMed: 16738102. 16738102 10.1099/ijs.0.64025-0

[79] Döbereiner J, Pedrosa FO (1987) Nitrogen-fixing bacteria in nonleguminous crop plants. Science Tech, Springer Verlag, Madison pp 1–155 (Brock/Springer series in contemporary bioscience).

[80] Huergo LF, Monteiro RA, Bonatto AC, Rigo LU, Steffens MBR et al. (2008) Regulation of nitrogen fixation in *Azospirillum* *brasilense* . In: Cassán FD GarciadeSalamone I Azospirillum sp.: cell physiology, plant interactions and agronomic research in Argentina. Asociación Argentina de Microbiologia, Argentina pp 17–36.

[81] Girvan MS, Bullimore J, Pretty JN, Osborn AM, Ball AS (2003) Soil type is the primary determinant of the composition of the total and active bacterial communities in arable soils. Appl Environ Microbiol 69: 1800-1809. doi:10.1128/AEM.69.3.1800-1809.2003. PubMed: 12620873. 12620873 10.1128/AEM.69.3.1800-1809.2003 PMC150080

[82] Brotonegro S (1974) Nitrogen fixation and nitrogenase activity in *Azotobacter* *chroococcum* . Commum Agric Univ 10:1-76.

[83] Christiansen-Weninger C, van Veen JA (1991) Nitrogen fixation by *Azospirillum* *brasiliense* in soil and the rhizosphere under controlled environmental conditions. Plant Breeding 12: 100-106.

[84] Zhang Y, Li D, Wang H, Xiao Q, Liu X (2006) Molecular diversity of nitrogen-fxing bacteria from the Tibetan Plateau,China. FEMS Microbiol Lett 260: 134–142. doi:10.1111/j.1574-6968.2006.00317.x. PubMed: 16842336. 16842336 10.1111/j.1574-6968.2006.00317.x

