# Supplementary figures and images for: Temporal Dynamics of Abundance and Composition of Nitrogen-Fixing Communities across Agricultural Soils
Source: PLoS One. 2013 Sep 13;8(9):e74500. doi: 10.1371/journal.pone.0074500 (PMC3772945; doi:10.1371/journal.pone.0074500)

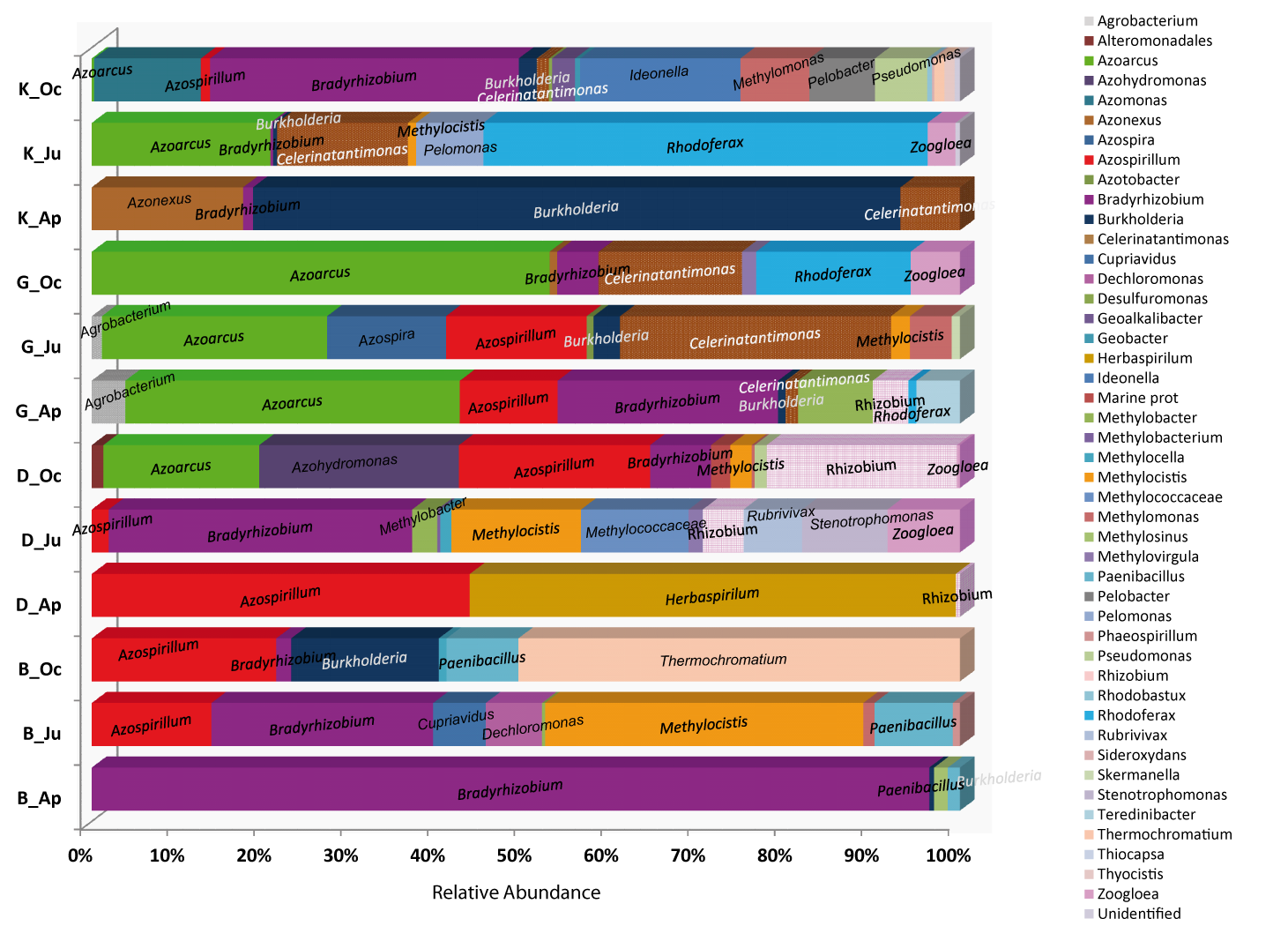

Supplement: Figure S4 — Taxonomic classification of the most abundant (“core”) nifH-gene sequences associated with the two sandy agricultural soils (B and D) at three sampling times, April, June and October. Multi-colored charts at the legend are shown for each sample correspondingly. (PNG) [file pone.0074500.s004.png]

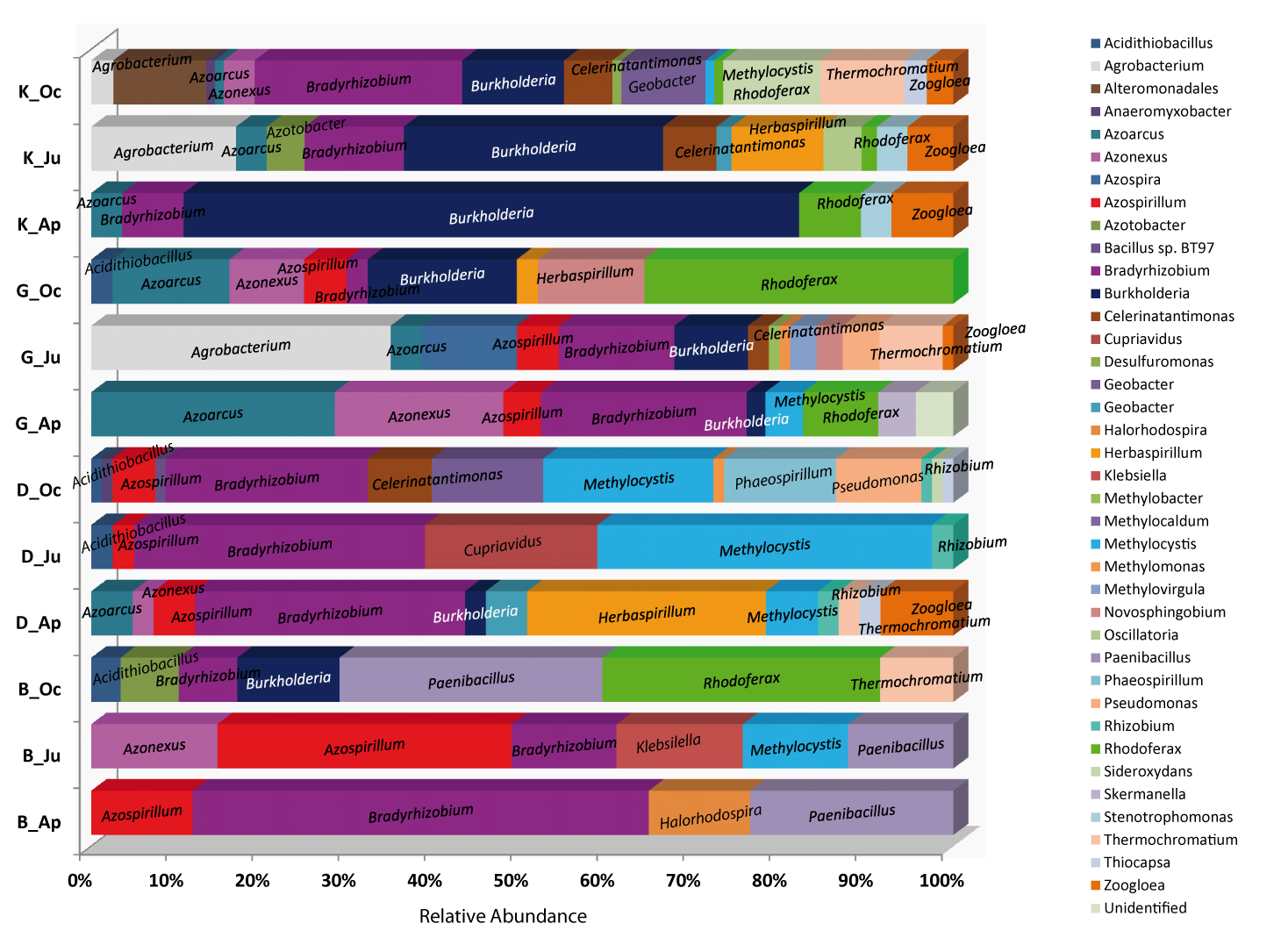

Supplement: Figure S5 — Taxonomic classification of the less abundant (“rare”) nifH-gene sequences associated with the two clayey agricultural soils (G and K) at three sampling times, April, June and October. Multi-colored charts at the legend are shown for each sample correspondingly. (PNG) [file pone.0074500.s005.png]
