# Supplementary material for: Temporal Dynamics of Abundance and Composition of Nitrogen-Fixing Communities across Agricultural Soils
Source: PLoS One. 2013 Sep 13;8(9):e74500. doi: 10.1371/journal.pone.0074500 (PMC3772945; doi:10.1371/journal.pone.0074500)
Supplement: Table S1 — Soil characteristics measured in this study. (DOCX) [file pone.0074500.s006.docx]

Table S1: Soil characteristics measured in this study.

| Abiotic parameter | Sampling time | Soils | | | |
| --- | --- | --- | --- | --- | --- |
|  |  | Buinen (B) | Droevendaal (D) | Kollumerward (K) | Grebbedijk (G) |
| pH | April | 4.20 | 5.00 | 7.40 | 7.20 |
|  | June | 4.60 | 5.10 | 7.40 | 7.00 |
|  | October | 4.40 | 4.70 | 7.40 | 7.40 |
|  | **Average** | 4.40 | 4.93 | 7.40 | 7.20 |
|  | **C.V.** | 0.05 | 0.04 | 0.00 | 0.03 |
| OM | April | 4.90 | 2.60 | 6.60 | 6.40 |
| (%) | June | 3.50 | 3.30 | 3.30 | 4.30 |
|  | October | 3.60 | 2.60 | 2.70 | 5.50 |
|  | **Average** | 4.00 | 2.83 | 4.20 | 5.40 |
|  | **C.V.** | 0.20 | 0.14 | 0.50 | 0.20 |
| Water content | April | 13.80 | 9.50 | 16.70 | 18.80 |
| (%) | June | 7.50 | 16.00 | 19.70 | 20.40 |
|  | October | 12.30 | 9.40 | 21.50 | 19.60 |
|  | **Average** | 11.20 | 11.63 | 19.30 | 19.60 |
|  | **C.V.** | 0.29 | 0.33 | 0.13 | 0.04 |
| N-NO_3_^-^ | April | 24.50 | 67.90 | 43.60 | 59.00 |
| (mg/kg) | June | 103.60 | 68.50 | 14.80 | 12.20 |
|  | October | 13.40 | 45.20 | 15.40 | 18.60 |
|  | **Average** | 47.17 | 60.53 | 24.60 | 29.93 |
|  | **C.V.** | 1.04 | 0.22 | 0.67 | 0.85 |
| N-NH_4_^+^ | April | 10.40 | 18.40 | 9.80 | 19.80 |
| (mg/kg) | June | 12.80 | 15.30 | 6.60 | 21.20 |
|  | October | 4.50 | 3.20 | 8.80 | 4.40 |
|  | **Average** | 9.23 | 12.30 | 8.40 | 15.13 |
|  | **C.V.** | 0.46 | 0.65 | 0.19 | 0.62 |

OM = organic matter; N-NO_3_^-^ = nitrate; N-NH_4_^+^ = ammonium.

Numbers are average of three replicates.
