# Supplementary material for: Temporal Dynamics of Abundance and Composition of Nitrogen-Fixing Communities across Agricultural Soils
Source: PLoS One. 2013 Sep 13;8(9):e74500. doi: 10.1371/journal.pone.0074500 (PMC3772945; doi:10.1371/journal.pone.0074500)
Supplement: Table S2 — PCR and cycling conditions for PCR-DGGE analysis and real time quantification of nifH gene. (DOCX) [file pone.0074500.s007.docx]

Table S2: PCR mixture and cycling conditions for PCR-DGGE analysis and real time quantification of bacterial 16S rRNA and *nif*H genes.

| **Primers DGGE (5’-3’)** | **PCR mixture** | **Thermal conditions** |  |
| --- | --- | --- | --- |
| **Total bacterial community**  *F968-GC*  *(*AACGCGAAGAACCTTAC)*  *R1401.1b*  (CGGTGTGTACAAGAC CCGGGAACG) | 0.2mM dNTPs, 3.75mM MgCl2, 1x buffer (Bioline), 1% formamide, 0.2µM each primer, 2.5U Taq polymerase (Bioline) | 95ºC 5 min  60ºC 1’ ( - 1º /cycle, until 55ºC); 72ºC 2 min 10 cycles  94ºC 1 min, 55ºC 1 min, 72ºC 2 min 20 cycles  Final extension of 72ºC 10 min |  |
| **N-fixing community**  *FPGH19* (TACGGCAARGGTGGNATHG)  *PolR* (ATSGCCATCATYTCRCCGGA)  *PolF-GC* * (TGCGAYCCSAARGCBGACTC)  *AQER* (GCCATCCATCTGTATGTCCA) | 0.20mM dNTPs, 1x buffer (Roche), 0.01mg BSA (20mg/ml), 0.5µM each primer, 0.5U Taq polymerase (Roche)  0.25mM dNTPs, 1x buffer (Roche), 0.01mg BSA (20mg/ml), 0.5µM each primer, 0.8U Taq polymerase (Roche) | 94°C, 5 min  94°C 60s, 56°C 1 min, 72°C 2min 30 cycles  Final extension of 72ºC 30 min  94°C, 5 min  94°C 60s, 48°C 1 min, 72°C 2min 30 cycles  Final extension of 72ºC 30 min |  |
| **Primers qPCR (5’-3’)** | **PCR mixtures** | **Thermal conditions** | |
| **Bacterial 16S rRNA gene**  *16SFP*  (GGTAGTCYAYGCMSTAAACG)  (Bach *et al*., 2002***)***  *16SRP* (GACARCCATGCASCACCTG) | 12.5µl Power Sybr Green PCR Master mix (Applied Biosystems), 0.5ul BSA (20mg/ml), 0.8µM each primer and 2ul DNA template | 95°C 10 min, 1 cycle  95°C for 27s, 62°C for 1 min, 72°C for 30s, 39 cycle | |
| ***nif*H gene**  *FPGH19* (TACGGCAARGGTGGNATHG)  *PolR* (ATSGCCATCATYTCRCCGGA) | 12.5µl Power Sybr Green PCR Master mix (Applied Biosystems), 0.5ul BSA (20mg/ml), 0.25µM each primer and 2ul DNA template | 95°C 10 min, 1 cycle  94°C for 60s, 55°C for 27s,  72°C for 60s, 39 cycle | |

*GC-clamp according to Muyzer et al., 1999
