# Supplementary material for: Temporal Dynamics of Abundance and Composition of Nitrogen-Fixing Communities across Agricultural Soils
Source: PLoS One. 2013 Sep 13;8(9):e74500. doi: 10.1371/journal.pone.0074500 (PMC3772945; doi:10.1371/journal.pone.0074500)
Supplement: Table S3 — PCR and cycling conditions for pyrosequencing of nifH gene. (DOCX) [file pone.0074500.s008.docx]

Table S3: PCR mixture and cycling conditions for pyrosequencing of *nif*H gene.

| **Primers (5’- 3’)** | **PCR mixtures** | **Thermal conditions** |
| --- | --- | --- |
| ***Specific primers:*** |  |  |
| *PolF*  (TGCGAYCCSAARGCBGACTC)  *PolR*  (ATSGCCATCATYTCRCCGGA) | 0.20mM dNTPs, 1x FastStart High Fidelity reaction buffer (Roche), 0.03mg BSA (20mg/ml), 0.5µM each primer, 0.25U FastStart High Fidelity enzyme (Roche), 50ng template DNA | 95°C, 5 min  94°C 1 min, 48°C 1 min, 72°C 1min 35 cycles  Final extension of 72ºC 10 min |
|  |  |  |
| ***Barcoded primers:*** |  |  |
| *RoeschF*  (ACCCGCCTGATCCTGCACGCCAAGG)  *RoeschR*  *(*ACGATGTAGATTTCCTGGGCCTTGTT) | 0.20mM dNTPs, 1x FastStart High Fidelity reaction buffer (Roche), 0.03mg BSA (20mg/ml), 0.5µM each primer, 0.25U FastStart High Fidelity enzyme (Roche), 1ul PCR with specific primers | 95°C, 5 min  94°C 45s, 50°C 45s, 72°C 45s 20 cycles  Final extension of 72ºC 10 min |
