# Supplementary material for: Temporal Dynamics of Abundance and Composition of Nitrogen-Fixing Communities across Agricultural Soils
Source: PLoS One. 2013 Sep 13;8(9):e74500. doi: 10.1371/journal.pone.0074500 (PMC3772945; doi:10.1371/journal.pone.0074500)
Supplement: Table S4 — Forward and reverse barcoded primers used for pyrosequencing of nifH gene. (DOCX) [file pone.0074500.s009.docx]

Table S4: Forward and reverse barcoded primers used for pyrosequencing of *nif*H gene.

| **Name** | **Key** | **MID** | **Specific Primer** | **Forward** |
| --- | --- | --- | --- | --- |
| MID_1A | CGTATCGCCTCCCTCGCGCCA | TCAG | ACGAGTGCGT | ACCCGCCTGATCCTGCACGCCAAGG |
| MID_2A | CGTATCGCCTCCCTCGCGCCA | TCAG | ACGCTCGACA | ACCCGCCTGATCCTGCACGCCAAGG |
| MID_15A | CGTATCGCCTCCCTCGCGCCA | TCAG | ATACGACGTA | ACCCGCCTGATCCTGCACGCCAAGG |
| MID_16A | CGTATCGCCTCCCTCGCGCCA | TCAG | TCACGTACTA | ACCCGCCTGATCCTGCACGCCAAGG |
| MID_17A | CGTATCGCCTCCCTCGCGCCA | TCAG | CGTCTAGTAC | ACCCGCCTGATCCTGCACGCCAAGG |
| MID_18A | CGTATCGCCTCCCTCGCGCCA | TCAG | TCTACGTAGC | ACCCGCCTGATCCTGCACGCCAAGG |
| MID_19A | CGTATCGCCTCCCTCGCGCCA | TCAG | TGTACTACTC | ACCCGCCTGATCCTGCACGCCAAGG |
| MID_20A | CGTATCGCCTCCCTCGCGCCA | TCAG | ACGACTACAG | ACCCGCCTGATCCTGCACGCCAAGG |
| MID_21A | CGTATCGCCTCCCTCGCGCCA | TCAG | CGTAGACTAG | ACCCGCCTGATCCTGCACGCCAAGG |
| MID_22A | CGTATCGCCTCCCTCGCGCCA | TCAG | TACGAGTATG | ACCCGCCTGATCCTGCACGCCAAGG |
| MID_23A | CGTATCGCCTCCCTCGCGCCA | TCAG | TACTCTCGTG | ACCCGCCTGATCCTGCACGCCAAGG |
| MID_24A | CGTATCGCCTCCCTCGCGCCA | TCAG | TAGAGACGAG | ACCCGCCTGATCCTGCACGCCAAGG |
|  | **Key** | **MID** | **Specific Primer** | **Reverse** |
| MID_33B | CTATGCGCCTTGCCAGCCCGC | TCAG | ATAGAGTACT | ACGATGTAGATTTCCTGGGCCTTGTT |
| MID_34B | CTATGCGCCTTGCCAGCCCGC | TCAG | CACGCTACGT | ACGATGTAGATTTCCTGGGCCTTGTT |
| MID_35B | CTATGCGCCTTGCCAGCCCGC | TCAG | CAGTAGACGT | ACGATGTAGATTTCCTGGGCCTTGTT |
| MID_36B | CTATGCGCCTTGCCAGCCCGC | TCAG | CGACGTGACT | ACGATGTAGATTTCCTGGGCCTTGTT |
| MID_37B | CTATGCGCCTTGCCAGCCCGC | TCAG | TACACACACT | ACGATGTAGATTTCCTGGGCCTTGTT |
| MID_38B | CTATGCGCCTTGCCAGCCCGC | TCAG | TACACGTGAT | ACGATGTAGATTTCCTGGGCCTTGTT |
| MID_39B | CTATGCGCCTTGCCAGCCCGC | TCAG | TACAGATCGT | ACGATGTAGATTTCCTGGGCCTTGTT |
| MID_40B | CTATGCGCCTTGCCAGCCCGC | TCAG | TACGCTGTCT | ACGATGTAGATTTCCTGGGCCTTGTT |
| MID_41B | CTATGCGCCTTGCCAGCCCGC | TCAG | TAGTGTAGAT | ACGATGTAGATTTCCTGGGCCTTGTT |
| MID_43B | CTATGCGCCTTGCCAGCCCGC | TCAG | TCGCACTAGT | ACGATGTAGATTTCCTGGGCCTTGTT |
| MID_44B | CTATGCGCCTTGCCAGCCCGC | TCAG | TCTAGCGACT | ACGATGTAGATTTCCTGGGCCTTGTT |
| MID_45B | CTATGCGCCTTGCCAGCCCGC | TCAG | TCTATACTAT | ACGATGTAGATTTCCTGGGCCTTGTT |
