# Supplementary material for: Temporal Dynamics of Abundance and Composition of Nitrogen-Fixing Communities across Agricultural Soils
Source: PLoS One. 2013 Sep 13;8(9):e74500. doi: 10.1371/journal.pone.0074500 (PMC3772945; doi:10.1371/journal.pone.0074500)
Supplement: Table S5 — Richness estimates and diversity indices for forward and reverse amplicon libraries at 90% similarity cutoff. (DOCX) [file pone.0074500.s010.docx]

Table S5: Richness estimates and diversity indices for forward and reverse amplicon libraries at 90% similarity cutoff.

| **Library** | **NS^a^** | **OTUs^b^** | **Estimated OTU richness** | | **Shannon^c^** | **ESC^d^** |
| --- | --- | --- | --- | --- | --- | --- |
|  |  |  | **Chao1** | **ACE** |  |  |
| ***Forward*** |  |  |  |  |  |  |
| B_Ap | 1921 | 40 | 48.25 (42.16; 71.48) | 53.53 (44.54; 80.31) | 2.09 (2.03; 2.15) | 0.95 |
| D_Ap | 2981 | 87 | 135.33 (105.92; 210.49) | 130.69 (107.37; 108.69) | 2.81 (2.75; 2.86) | 0.93 |
| K_Ap | 3530 | 80 | 119 (94.76; 183.05) | 123.4 (99.41; 177.03) | 2.53 (2.47; 2.58) | 0.91 |
| G_Ap | 3313 | 71 | 102.67 (81.39; 167.45) | 99.46 (82.48; 141.56) | 2.66 (2.61; 2.71) | 0.92 |
| B_Ju | 2071 | 44 | 65 (50.07; 116.68) | 59.55 (49.57; 87.45) | 1.98 (1.92; 2.04) | 0.95 |
| D_Ju | 2779 | 65 | 78.6 (69.29; 108.09) | 89.17 (74.59; 125.92) | 2.66 (2.61; 2.72) | 0.93 |
| K_Ju | 5141 | 118 | 224 (168.11; 342.25) | 256.19 (192.21; 375.32) | 2.83 (2.79; 2.88) | 0.88 |
| G_Ju | 5892 | 110 | 196.67 (146.63; 315.03) | 174.71 (142.76; 223.78) | 2.93 (2.89; 2.97) | 0.86 |
| B_Oc | 2266 | 63 | 110.25 (80.87; 187.87) | 134.79 (95.36; 222.26) | 2.58 (2.53; 2.64) | 0.95 |
| D_Oc | 3671 | 120 | 183.58 (150.01; 254.72) | 193.92 (160.06; 256.39) | 2.98 (2.94; 3.03) | 0.91 |
| K_Oc | 4171 | 107 | 164 (131.98; 237.07) | 173 (141.24; 237.01) | 2.94 (2.89; 2.99) | 0.90 |
| G_Oc | 3669 | 74 | 108.5 (86.41; 169.855) | 106.57 (88.35; 147.91) | 2.47 (2.43; 2.52) | 0.91 |
|  |  |  |  |  |  |  |
| ***Reverse*** |  |  |  |  |  |  |
| B_Ap | 1916 | 39 | 124.50 (63.95; 131.96) | 112.53 (62.16; 272.43) | 2.16 (2.11; 2.22) | 0.96 |
| D_Ap | 3346 | 80 | 122.16 (94.52; 202.49) | 109.53 (92.31; 150.81) | 2.88 (2.83; 2.93) | 0.93 |
| K_Ap | 3791 | 82 | 136 (102.11; 227.01) | 123.41 (100.33; 175.56) | 2.82 (2.78; 2.86) | 0.92 |
| G_Ap | 3311 | 67 | 94.14 (76.04; 148.51) | 95.25 (78.69; 135.26) | 2.49 (2.45; 2.54) | 0.93 |
| B_Ju | 2317 | 37 | 53.5 (41.25; 100.95) | 55.29 (42.96; 93.12) | 2.09 (2.04; 2.15) | 0.95 |
| D_Ju | 3416 | 72 | 91 (78.30; 129.34) | 91.40 (80.04; 118.82) | 2.62 (2.57; 2.67) | 0.92 |
| K_Ju | 5372 | 94 | 146.8 (115.49; 223.69) | 172.97 (131.78; 259.11) | 2.99 (2.96; 3.03) | 0.88 |
| G_Ju | 6328 | 101 | 157.1 (124.04; 237.63) | 156.32 (127.74; 215.48) | 2.84 (2.80; 2.88) | 0.86 |
| B_Oc | 2422 | 40 | 85.5 (52.08; 211.299) | 65.88 (48.46; 119.12) | 2.34 (2.29; 2.39) | 0.95 |
| D_Oc | 3845 | 82 | 183.5 (117.14; 375.17) | 138.76 (106.86; 211.57) | 2.91 (2.87; 2.96) | 0.91 |
| K_Oc | 4944 | 101 | 146.77 (120.43; 208.8) | 159.08 (129.79; 218.16) | 2.99 (2.95; 3.03) | 0.89 |
| G_Oc | 3760 | 57 | 109.5 (73.71; 221.96) | 99.61 (73.62; 166.27) | 2.37 (2.33; 2.420 | 0.92 |

^a^Number of sequences in each library.

^b^Calculated with DOTUR at the 10% distance level.

^c^Shannon diversity index calculated using DOTUR (10% distance level).

^d^Estimated sample coverage: Cx = 1 - (Nx/n), where Nx is the number of unique sequences and n is the total number of sequences.

Values in brackets are 95% confidence intervals as calculated using DOTUR.
